# EnhancerNet: a predictive model of cell identity dynamics through enhancer selection

**DOI:** 10.1242/dev.202997

**Published:** 2024-10-09

**Authors:** Omer Karin

**Affiliations:** Department of Mathematics, Imperial College London, London, SW7 2AZ, UK

**Keywords:** Cell fate, Cell identity, Dynamical systems, Enhancer selection, Statistical physics, Systems biology

## Abstract

Understanding how cell identity is encoded by the genome and acquired during differentiation is a central challenge in cell biology. I have developed a theoretical framework called EnhancerNet, which models the regulation of cell identity through the lens of transcription factor-enhancer interactions. I demonstrate that autoregulation in these interactions imposes a constraint on the model, resulting in simplified dynamics that can be parameterized from observed cell identities. Despite its simplicity, EnhancerNet recapitulates a broad range of experimental observations on cell identity dynamics, including enhancer selection, cell fate induction, hierarchical differentiation through multipotent progenitor states and direct reprogramming by transcription factor overexpression. The model makes specific quantitative predictions, reproducing known reprogramming recipes and the complex haematopoietic differentiation hierarchy without fitting unobserved parameters. EnhancerNet provides insights into how new cell types could evolve and highlights the functional importance of distal regulatory elements with dynamic chromatin in multicellular evolution.

## Teaser

**Summary:** EnhancerNet, a model for cell identity based on TF-enhancer interactions, explains diverse phenomena and makes specific predictions on large-scale cell identity processes, including differentiation, reprogramming and the evolution of multicellularity.

## INTRODUCTION

A single fertilised animal egg cell divides and differentiates into many cell types that are maintained throughout the life of the organism. Each cell type is associated with a distinct gene expression profile, which can be acquired by gradual differentiation through progenitor states or by reprogramming. Despite great advancements in the molecular characterization of cell dynamics, many fundamental questions regarding how cell types are acquired and maintained remain unanswered. Specifically, key open challenges include how cell types and their associated expression profiles are encoded by the genome, identifying the regulatory mechanisms underlying stepwise differentiation versus direct reprogramming pathways, understanding how signalling triggers the acquisition of particular cell identities and determining how new cell types with unique expression signatures can evolve.

Addressing these questions requires formulating quantitative hypotheses on the dynamics of gene expression over time and in response to perturbations. From a theoretical perspective, cell types correspond to stable attractors of the gene regulatory network of the cell (Waddington, 1957; Huang et al., 2005; Lang et al., 2014; Teschendorff and Feinberg, 2021); that is, the gene regulatory network has multiple stable configurations. These stable configurations are set by feedback interactions between hundreds of transcription factors (TFs), which are DNA-binding proteins that can modulate the expression of other genes. Specific cell types are associated with the expression of distinct combinations of TFs, and TFs play a crucial role in maintaining and modulating cell identity (Burke et al., 1995; Heinz et al., 2010; Holmberg and Perlmann, 2012; Hnisz et al., 2013; Whyte et al., 2013; Kelaini et al., 2014; Saint-André et al., 2016; Reiter et al., 2017; Reilly et al., 2020; Hobert, 2021; Wang et al., 2021; Almeida et al., 2021).

The importance of TFs is reflected in their central role in models of cell identity dynamics. Early models focused on how small transcriptional network motifs, involving only a few TFs, can generate cell fate bifurcations, such as those specific to blood lineages and early embryogenesis (Ferrell, 2002; Huang et al., 2007; Graham et al., 2010; Bessonnard et al., 2014; Sáez et al., 2022). Although these models are important for understanding gene expression dynamics at specific decision points, they cannot predict large-scale dynamics of the cell identity network, such as complex, hierarchical differentiation patterns or reprogramming between distinct cell types.

In contrast to these ‘small network’ models, pioneering work has demonstrated that aspects of cell identity behaviour can be captured by high-dimensional attractor models based on Classical Hopfield networks (Lang et al., 2014; Fard et al., 2016; Pusuluri et al., 2017; Guo and Zheng, 2017; Teschendorff and Feinberg, 2021; Yampolskaya et al., 2023; Smart and Zilman, 2023). Classical Hopfield networks are well-established models for dynamical systems that encode combinatorial attractor states (patterns) through the interactions of their components (Amari, 1972; Little, 1974; Hopfield, 1982). In the context of transcriptional networks, the state of the dynamics is represented by the activity of cell identity TFs, with the attractor states corresponding to distinct cell types. In Hopfield networks, the entire dynamics of the system can be specified solely by defining the attractor states, which are the observed cell types. Although in their original formulation Classical Hopfield networks cannot encode for correlated expression profiles, this limitation can be overcome by an orthogonal projection of the expression profiles (Amit, 1989; Lang et al., 2014). This allows for predictions about the gene regulatory network dynamics based on mere knowledge of the cell types encoded by the network. Lang et al. exploit this property to demonstrate that it is possible to capture and predict the effects of TF overexpression on cellular reprogramming (Lang et al., 2014). The Hopfield formulation also predicts that mixtures of attractor states may themselves be attractor states (known as ‘spurious attractors’). Lang et al. propose that this phenomenon explains the observed expression profiles of partially reprogrammed cells (Lang et al., 2014).

Although this work provides a compelling motivation for applying attractor networks to study cell identity dynamics, it has several important limitations that restrict its applicability. First, although Hopfield dynamics provide an attractive computational model, the relation between this model and underlying molecular mechanisms for cell type specification is unclear. The model also specifies binary expression patterns and does not capture continuous changes in expression levels, such as low-level multilineage priming in progenitor states, which is a hallmark of hierarchical differentiation (Hu et al., 1997; Miyamoto et al., 2002; Mercer et al., 2011; Nimmo et al., 2015; Kim et al., 2016; Olsson et al., 2016; Briggs et al., 2018; Zheng et al., 2018; Martin et al., 2021; Singh et al., 2022). Finally, such progenitor states must be explicitly specified in the model as attractor cell types, rather than emerging naturally from the dynamics.

Here, I develop a theoretical framework for modelling the dynamics of the cell identity network by considering the feedback regulation of TFs through enhancers, which I denote as EnhancerNet. Enhancers are regulatory elements that are crucial for cell identity specification. TFs bind to enhancers to activate cell type-specific expression patterns. Each enhancer can be bound by multiple TFs and, in turn, initiate gene expression in one or more distal target genes (Pennacchio et al., 2013; Uyehara and Apostolou, 2023). The binding of TFs to an enhancer recruits co-factors that can alter its activity by modulating epigenetic properties, such as the biochemical characteristics of its associated chromatin. This, in turn, affects the transcription initiation rate associated with the enhancer (Heintzman et al., 2009; Creyghton et al., 2010; Calo and Wysocka, 2013; Park et al., 2021; Hansen et al., 2022).

A well-documented experimental observation reveals a symmetry between TF binding and enhancer regulation (Hnisz et al., 2013; Whyte et al., 2013; Adam et al., 2015; Saint-André et al., 2016; Feng et al., 2023). The TFs that determine cell identity bind to enhancers that regulate both their own expression and that of other identity-determining TFs that are co-expressed in the same cell types. This interaction forms dense autoregulatory networks of TF-enhancer interactions. These dense autoregulatory networks likely play a crucial role in cell type specification, as their associated enhancers exhibit activation patterns that are highly cell-type specific (Hnisz et al., 2013; Heinz et al., 2015). The cell type specificity of enhancer activity contrasts with the broader activity of TFs, which may be shared across multiple cell types (Hnisz et al., 2013).

I show that the symmetry between TF binding and enhancer regulation imposes an exacting constraint on the dynamics of the cell identity network model, resulting in a highly simplified dynamical model that is predictive and captures broad experimental observations on cell type specification, enhancer selection, differentiation through progenitors, and reprogramming, without the need of fitting unobserved parameters. I demonstrate a mathematical analogy between our mechanistic model and Modern Hopfield networks (Krotov and Hopfield, 2016; Ramsauer et al., 2020 preprint) that overcomes the limitations of previous approaches. This analogy provides a mechanistic link between models for associative memories and cell identity networks, explains the role of distal regulatory elements with dynamic chromatin in multicellular evolution, and provides specific and testable predictions.

## RESULTS

### Model for enhancer activation dynamics in transcriptional feedback networks

**Fig. 1.**
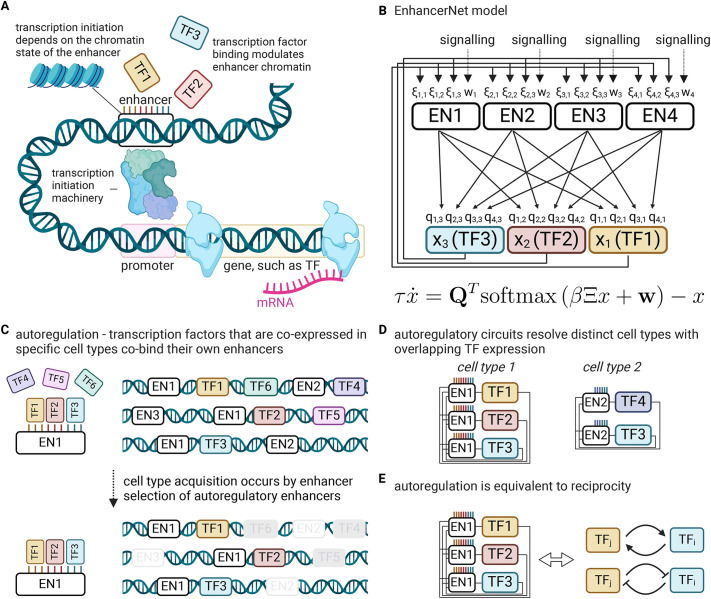
**Model for the regulation of cell identity transcription factors by enhancers.** (A) Enhancers are *cis*-regulatory elements that can initiate transcription in distant genes through interaction with specific transcription factors (TFs) and transcriptional machinery. Each enhancer can interact with several TFs, and each TF can be controlled by multiple enhancers. Binding of TFs modulates enhancer chromatin and can increase the transcription initiation rate of the enhancer. (B) Enhancer types (top) bind specific combinations of TFs (bottom) according to binding strengths specified by the 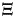 matrix, and, in turn, initiate transcription according to the rate matrix 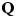. The weight vector 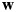 determines baseline activity and may be modulated by binding of signalling TFs. (C) Autoregulation is the observation that TFs that are co-expressed in specific cell types co-bind their own enhancers. These enhancers are, in turn, selected and activated in these specific cell types. (D) Different cell types are associated with specific enhancers that may have overlapping TF binding. (E) Autoregulation constrains 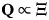 and implies reciprocity.

I began by deriving a general mathematical model for the feedback regulation of TF expression through their interaction with enhancers (Fig. 1A). Enhancers consist of multiple TF binding motifs (Spitz and Furlong, 2012). The binding of TFs potentiates enhancer activity by recruiting transcriptional co-factors. These co-factors modulate the biochemical properties of enhancer chromatin through processes such as histone acetylation, leading to the recruitment of transcription initiation factors and ultimately resulting in the transcription of enhancer-associated genes (Narita et al., 2021; Panigrahi and O'Malley, 2021).

To capture these complex molecular mechanisms within an effective mathematical framework, I considered the following setup. An enhancer (indexed *i*) is characterized by two vectors: a vector, 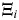, denoting its binding affinity to different identity TFs (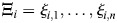); and a vector, *Q*_*i*_, denoting the rates at which it can induce the transcription of the same TFs (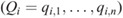). The binding of TFs to an enhancer modulates the state of the enhancer chromatin, which in turn sets the rate at which the transcriptional initiation machinery is recruited to the enhancer. Specifically, I assumed that the chromatin state sets the energy for the recruitment of the transcription initiation machinery and that, on the timescale of cell identity changes, the recruitment rate is captured by an equilibrium distribution (see Materials and Methods). Taken together, the dynamics of the gene regulatory network are given by:
(1)

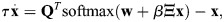
where 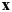 is the vector of TF expression whose entries are *x*_*i*_, 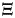 is a matrix whose entries are *ξ*_*i*,*j*_, **Q** is the association matrix whose entries are *q*_*i*,*j*_, *τ* is the timescale of the dynamics and *β* is an effective inverse temperature parameter that depends on the turnover of chromatin modifications (see Materials and Methods, Fig. 1B). The baseline activity vector 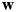 depends on effects upstream of the cell identity network, such as the binding of effector TFs for signalling pathways (Hnisz et al., 2015). In this study, I will refer to the network model and its associated dynamics as EnhancerNet.

Although Eqn 1 considered different physical enhancers as separate entities, in practice, enhancers with similar binding profiles can appear throughout the genome (Saint-André et al., 2016; Kvon et al., 2021), corresponding to similar rows of 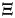. I denote these as enhancer types and note that Eqn 1 can be rewritten so that 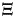 is a matrix of enhancer types (i.e. with distinct rows) and the matrix 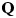 corresponds to the overall association between enhancer types and TF expression (see Materials and Methods). Hereafter, I will assume that Eqn 1 captures the dynamics of enhancer types, which can correspond to the activation of multiple physical enhancers.

### Autoregulation of TF-enhancer interactions constrains the EnhancerNet model

Eqn 1 can be further constrained by taking into account well-established properties of developmental networks. TFs associated with specific cell identities form densely interconnected networks in which they co-bind adjacent enhancers, which in turn activate the same TFs (Whyte et al., 2013; Hnisz et al., 2013, 2015; Saint-André et al., 2016) (Fig. 1C,D). This implies a strong positive correlation between the rows of 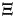, which correspond to binding profiles of enhancers, and the rows of 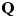, which correspond to the effect of these enhancers on TF transcription. This was captured by taking 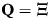 (Fig. 1E; relaxing this to assume that 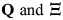 are merely correlated does not affect our conclusions). This gives the following model for the dynamics:
(2)

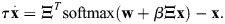
The dynamics captured by Eqn 2 are mathematically similar to models for efficient memory storage in artificial neural networks (Ramsauer et al., 2020 preprint). This relationship can be exploited when analysing various properties of the dynamics. These dynamics are associated with a potential function (or ‘potential landscape’) 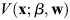 (see Materials and Methods). The shape of this potential landscape determines how gene expression changes over time, including the stable gene expression patterns associated with different cell identities, which correspond to minima of the potential landscape.

Autoregulation, captured by 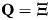, implies that the interactions between TFs in the developmental network are reciprocal, i.e. for each pair of TFs *k*, *j*, the effect of increasing TF *j* on the expression rate of TF *k* is the same as the effect of increasing TF *k* on the expression rate of TF *j* (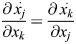). Moreover, reciprocity and autoregulation are effectively equivalent (see Materials and Methods) (Fig. 1E). This equivalence explains the observation that reciprocal interactions are ubiquitous in developmental networks (Kraut and Levine, 1991; Milo et al., 2002; Alon, 2007; Huang et al., 2007).

Reciprocal interactions between TFs can be positive (mutual activation) or negative (mutual repression), with both possibilities common in developmental networks. This is also captured in Eqn 2, despite the fact that, in the model, enhancers only enhance transcription, and the binding of TFs to enhancers increases their activity. Repression in the model is a by-product of global inhibition by competition over shared transcriptional machinery.

### Robust specification of combinatorial cell types through interaction between TFs and enhancers

**Fig. 2.**
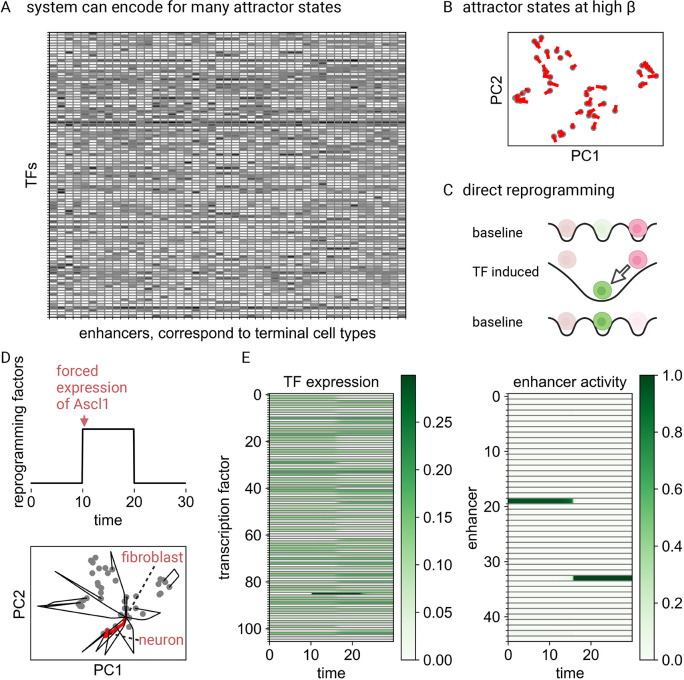
**Specification of cell types and direct reprogramming.** (A) The EnhancerNet model was initialized with the TF expression profiles of 45 cell types from Tabula Muris, taking 105 transcription factors (TFs) with high variability between cell types (see Materials and Methods). The resulting 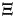 matrix (transposed) is plotted, with larger values corresponding to darker colours. (B) At high *β* each row of 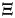 is an attractor of the network. PCA transformation of 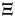 is shown, with transformed individual rows, corresponding to enhancer-binding patterns, plotted in grey. They are all attractor states – trajectories that start in the vicinity of each of these states converge to them. Red lines correspond to trajectories starting from rows of 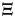 with 25% multiplicative noise. (C) Constitutive overexpression of TFs alters the attractor landscape and can result in a bifurcation where cell types lose stability and transition to another cell type. Arrow indicates transition between gene expression states. (D) The overexpression of Ascl1 transitions fibroblasts to neurons in the model, capturing the known effect of Ascl1 overexpression in fibroblasts. The reprogramming path is plotted in red (lower panel), with black paths capturing other known reprogramming recipes (see Materials and Methods). (E) Expression of TFs (left panel) and enhancer activity (right panel), corresponding to the probability that transcription is initiated from the enhancer.

Cell identity is established through the process of enhancer selection (Heintzman et al., 2009; Hnisz et al., 2013, 2015; Saint-André et al., 2016). In this process, cell type-specific enhancers are marked for activation, leading to the expression of a distinct pattern of transcription factors associated with these enhancers. This is recapitulated in our model when the inverse temperature parameter *β* is sufficiently large (see Materials and Methods). Under this condition, the cell types encoded by the gene regulatory network, which represent stable attractors for the dynamics, are given by the rows of the enhancer-associated matrix 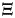 (or, in the general case, by the rows of 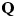; see Materials and Methods). Specifically, the stable fixed points of the network are represented by the vectors (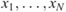)=(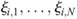) for all enhancer types (Fig. 2A,B). Each stable fixed point corresponds to a cell type with a unique transcription factor expression pattern. At these fixed points, cell type-specific enhancers exhibit maximal activity. These fixed points are robust to a large degree of asymmetry in TF-enhancer interactions (Fig. S1).

Signalling activity can induce specific cell fates or restrict inducible cell fates (Perrimon et al., 2012; Grover et al., 2014; Hnisz et al., 2015). This phenomenon is recapitulated in our model through the effect of signalling on 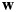, which in turn modulates the basins of attraction for each cell type. Signals that increase the activity of specific enhancers cause the cell types associated with these enhancers to become accessible from a broader range of gene expression states. As discussed later in the context of differentiation, this provides a mechanism to control the relative production of different cell types. Conversely, states with weak induction may become destabilized and thus reprogrammed to an alternative cell type. Importantly, the effect of signalling through changes in 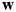 does not alter the TF identity of the cell types. Thus, the model provides a mechanism for how signalling can control cell type production without interfering with cell identities.

From a modelling perspective, the observation that cell types correspond to rows of 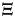 suggests an intriguing possibility: that Eqn 2 can be parameterized using the TF identities of the observed (terminal) cell types, which can be retrieved from gene expression datasets. This leaves the inverse temperature parameter *β* and the signalling vector 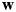 as the only free parameters. As I will show, in a variety of settings, there are strong constraints on both parameters. The model can thus provide predictions on the dynamics of the entire transcriptional network by considering only the observed cell types. In the rest of this article, I will scrutinize this possibility and demonstrate that, using this approach, the complex dynamics of the regulatory network can indeed be reconstructed in a variety of settings.

### EnhancerNet recapitulates direct reprogramming between cell types and predicts reprogramming recipes

I next tested whether the EnhancerNet model could predict the dynamics of the regulatory network controlling cell identity. Our analysis focused on the two broad classes of cell identity change: direct reprogramming, in which there are direct transitions between cell types; and hierarchical differentiation processes that start from (or transition through) multipotent progenitor states.

Direct reprogramming can be experimentally carried out by overexpressing TFs (Wang et al., 2021). Direct reprogramming has been demonstrated between dozens of cell types by overexpressing a wide range of TFs and TF combinations. Two clear distinguishing features for whether specific TFs can efficiently reprogram into a target cell type are that these factors are: (1) highly expressed in the target cell type and (2) their expression is unique to that cell type (D'Alessio et al., 2015).

The very existence of direct reprogramming between distantly related cell types is beyond the scope of classical models for cell fate bifurcations that are based on competition between a small set of lineage-determining factors. However, it is a straightforward consequence of the dynamics of Eqn 2 (Fig. 2C). The overexpression of the TF *x*_*j*_ modulates the basins of attraction of each cell type, which can then encompass other (previously stable) cell type expression patterns. If a cell has initially been placed in one of these distant patterns, i.e. 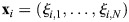, then, after TF overexpression, it can transition directly to a new pattern: 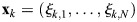.

Which transcription factors are most efficient in reprogramming to a specific cell type? The optimal transcription factor combination will uniquely increase the basin of attraction of the target cell type, while avoiding increasing the basins of attraction of other cell types. The degree of increase in the basin of attraction of cell type *k* by the overexpression of TF *j* is proportional to its binding association *ξ*_*k*,*j*_, which also corresponds to its expression in the target cell type (see Materials and Methods). Thus, TFs that are effective for reprogramming to a given cell type are predicted by the model to have high expression in that cell type and that this high expression is unique to the target cell type. This captures the known properties of reprogramming factors and is consistent with computational methods to identify reprogramming factors (D'Alessio et al., 2015; Rackham et al., 2016).

To test whether the model can recapitulate known reprogramming recipes, I generated a dataset for 45 cell identities using Tabula Muris (Schaum et al., 2018) (see Materials and Methods, Fig. 2A). I then set 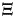 according to the TFs used in 12 established reprogramming recipes, as well as other TFs with high expression and variability. The transient overexpression of the reprogramming factors recapitulated the known reprogramming behaviour, with the system transitioning from the original attractor state (e.g. fibroblast) to an end attractor state, in line with the experiment (Fig. 2D,E). Thus, the EnhancerNet model can quantitatively recapitulate direct reprogramming, with no fitting parameters and by only considering the observed cell types.

As a specific demonstration of direct reprogramming, consider the reprogramming of a fibroblast into a neuron by overexpression of Ascl1 (Fig. 2E) (Chanda et al., 2014). The cell begins in a fibroblast state, where a specific enhancer type is strongly activated. The transient activation of a specific TF can destabilise this state, resulting in a transition to a neuron state that is associated with the activation of a neuron-specific enhancer type. The dynamics of this transition are one-dimensional and, in the presence of noise, correspond to a stochastic barrier crossing between two potential minima, which is consistent with previous quantitative analyses (Fig. S2) (Pusuluri et al., 2017).

### EnhancerNet recapitulates hierarchical differentiation dynamics and predicts progenitor identity

Direct reprogramming is an important experimental phenomenon and may also occur in natural settings after tissue perturbation (Merrell and Stanger, 2016). In homeostatic and developmental settings, however, it is more typical for differentiation trajectories to occur through a series of multipotent progenitor states (Moris et al., 2016). Such differentiation trajectories have been extensively studied in mammalian haematopoiesis, where a population of multipotent progenitors can give rise to many blood lineages (Månsson et al., 2007; Orkin and Zon, 2008). Other examples include the intestinal epithelium (Kim et al., 2016; Singh et al., 2022), stomach epithelium (Qiao et al., 2007) and skin (Toma et al., 2005), as well as throughout development (Briggs et al., 2018).

Differentiation trajectories share several common characteristics. Differentiation proceeds in a directed manner through a series of progenitor states. Each progenitor may acquire one of several terminal fates, with the range of target cell fates becoming more restricted as differentiation proceeds. For example, the cell may initially be in a multipotent progenitor state, and then transition to a bipotent state followed by a unipotent state. These dynamics are most famously conveyed by the Waddington landscape (Waddington, 1957), with the image of a ball rolling down a hill segregated by valleys capturing the progressive restriction of cell fate as it progresses through transitional progenitor states. Progenitors co-express at low levels the lineage-determining TFs associated with their target fates, which is a phenomenon known as multilineage priming (Hu et al., 1997; Olsson et al., 2016; Briggs et al., 2018; Zheng et al., 2018; Martin et al., 2021). Although the Waddington landscape image has gained great popularity for conceptualizing the dynamics of cell fate specification and for developing quantitative models for differentiation dynamics (Zhou et al., 2012; Rand et al., 2021), it is not clear how these complex hierarchical dynamics are implemented by the gene regulatory network.

**Fig. 3.**
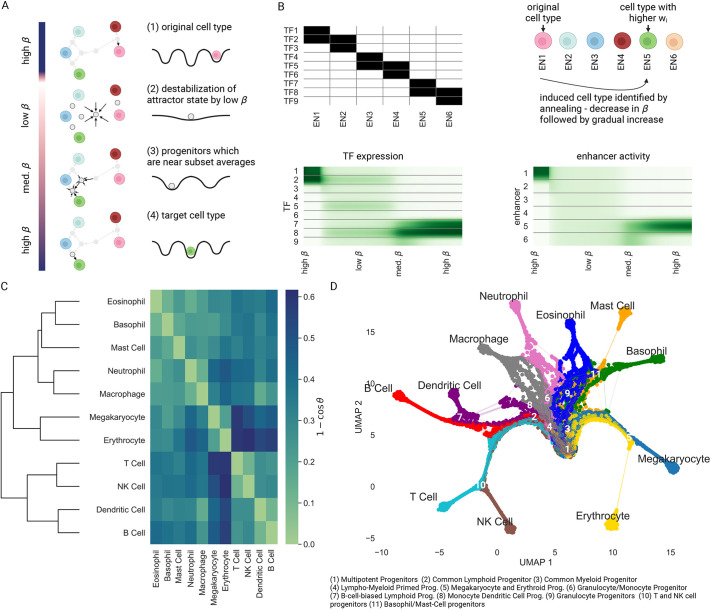
**Hierarchical differentiation by annealing.** (A) Annealing is the process by which *β* is transiently decreased and then increased. The decrease in *β* transitions the cell to a global attractor state corresponding to a multipotent progenitor with an averaged expression profile of terminal cell types. As *β* increases again, new attractor states are created, which are averages of smaller subsets of the terminal cell types, corresponding to progenitor states with limited potency until, finally, the cell transitions to a terminal cell type. (B) Progenitor states appear in subsets of cell types that have high cosine similarity. In this example, I considered a simple setup where enhancers bind overlapping transcription factors (TFs) (top left, with positive TF binding indicated in black). The dynamics begin from a cell type corresponding to the binding profile of EN1, and EN5 has a slight positive weight *w*_5_=0.05. Annealing was simulated as in the top right schematic. The dynamics proceed through progenitors that show multilineage priming (bottom left) and transition from widespread enhancer activity to cell type-specific activity (bottom right). (C) The cosine similarity of the expression profiles of the terminal blood lineages, where expression profiles were calculated using the TFs that show both sufficiently high interlineage variability (see Materials and Methods). Hierarchical clustering according to cosine similarity recreates the ‘classical’ haematopoiesis differentiation tree. (D) UMAP plot of differentiation trajectories, generated by direct simulation of annealing in an EnhancerNet model calibrated by the haematopoietic terminal lineage expression profiles. Trajectories were simulated from an initial homogeneous population with the addition of noise and with 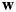 adjusted to produce a balanced differentiation profile (see Materials and Methods). Points represent samples of the trajectories at constant time intervals, with the colour corresponding to the identity of the final state. Simulation recreates the observed progenitor states in haematopoiesis, including deviations from tree-like differentiation.

Here, I show that Waddingtonian cell fate specification dynamics and the existence and identity of the progenitor states is an emergent property of the dynamics captured by Eqn 2. These are due to the second mechanism for cell-type transitions in the model, annealing, where *β* is transiently decreased by the cell (‘heating up’) and then slowly increased (‘cooling down’) (Fig. 3A). Recall that *β* is a coarse-grained parameter that depends on the regulation of chromatin by TF binding, and can be controlled by molecular mechanisms. Decreasing *β* results in a widespread pattern of enhancer activation and gene expression, while increasing *β* results in the activation of more specific enhancers, in line with experimental knowledge of differentiation hierarchies (Gulati et al., 2020). Specifically, decreasing *β* destabilises terminal attractor states, while increasing *β* restabilises them, resulting in a transition towards a new cell type (Fig. 3).

The annealing process proceeds as follows. The accessible and induced cell types, given by the rows of 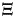, where 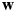 is sufficiently large, are all stable when *β* is very large, while their global average is the only stable state when *β* is small. This state expresses at low levels the TFs associated with all target lineages, and it corresponds to a multi-potent progenitor cell identity. As *β* increases, this state loses stability, and new stable states appear transiently. These new states are averages of increasingly restricted subsets of cell types, and they correspond to progenitors of restricted potential. Annealing thus recapitulates the hierarchical Waddingtonian differentiation dynamics and low-level multilineage progenitor expression patterns.

While progenitors generally correspond to averages of terminal cell types, not all averages are equally likely to be observed as stable progenitors. Rather, the stable progenitor states correspond to subsets of cell types with high internal similarity in their expression profiles (measured by cosine distance). As an illustrative example, I considered a simulation of a regulatory network with nine TFs, indexed 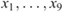, and six enhancers, indexed 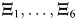. Each enhancer can be bound by two TFs, and enhancers 1-2, 3-4 and 5-6 overlap by a single TF (Fig. 3B). The initial state corresponded to an expression profile associated with 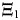. I also take a slightly larger weight *w*_5_ for 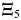, corresponding to induction of this state by signalling. Annealing (a decrease in *β* followed by a gradual increase) transitions the cell through two progenitors: a ‘multipotent progenitor’ (low *β*), which corresponds to the average expression of all the enhancers; and a ‘restricted potential progenitor’ (intermediate *β*) that is associated with the average expression of 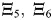 and, finally, differentiation to 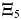. Thus, differentiation occurs through Waddingtonian dynamics associated with multilineage priming, allowing transition to an induced cell fate.

The model makes the specific prediction that differentiation hierarchies, including the identity of observed progenitor states, can be estimated by only knowing the identities of the terminal cell states. The model specifically predicts that they will appear only between transcriptional profiles with high internal cosine similarity. To test this, I considered differentiation in haematopoiesis, a system in which differentiation trajectories are complex and have been extensively studied. Using data on haematopoietic lineages in mice (extracted from Haemopedia; Choi et al., 2019), I considered the expression profile of all TFs that showed variability between terminal lineages and used the model to estimate the differentiation hierarchy that generated these lineages.

I first tested the hypothesis that progenitors emerge between correlated expression profiles by performing hierarchical clustering according to pairwise cosine similarity between the expression profiles of terminal cell types (Fig. 3C). Hierarchical clustering provides a heuristic approach to estimate the predicted identity of progenitor states from the terminal expression profiles. It produces a dendrogram (tree) that shows how expression profiles can be progressively grouped into clusters based on their similarity. This resulted in a tree structure that recreates the classic haematopoietic hierarchy (Fig. 3C). Branching points at the dendrogram capture the common lymphoid progenitor (CLP), common myeloid progenitor (CMP), megakaryocyte and erythroid progenitor (MEP), granulocyte and macrophage progenitor (GMP), T and NK cell progenitors (TNK), and B cell-biased lymphoid progenitor (BLP). Thus, a simple unsupervised clustering method with access to only the terminal cell fate information can reproduce a faithful estimate of the known complex differentiation landscape in haematopoiesis.

Motivated by the effectiveness of hierarchical clustering in predicting progenitor states, I developed a computational approach to model differentiation into multiple cell types. This approach is based on annealing in Eqn 2. Annealing trajectories were simulated beginning with a homogeneous initial population representing haematopoietic stem cells. To account for natural variability in differentiation paths, noise was introduced to Eqn 2 (our findings remained robust across various noise magnitudes, Fig. S3). In physiological settings, the production rate of each cell type is regulated by negative feedback, where the terminal cell population size inhibits its own production. This is exemplified by the negative-feedback regulation of red blood count through EPO signalling (Grover et al., 2014). To incorporate this regulation, I implemented a negative-feedback routine to tune 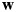, allowing each cell type to inhibit its own production (see Materials and Methods). This resulted in dynamics that generated all cell fates in a balanced manner. The differentiation trajectories were then visualized on a UMAP plot (Fig. 3D), revealing that differentiation dynamics proceed through a series of progenitors corresponding to those observed in haematopoiesis. Thus, our model quantitatively recapitulates differentiation in haematopoiesis without requiring parameter fitting.

Although the heuristic approach of hierarchical clustering produces a tree-like structure that recapitulates the main progenitor states in haematopoiesis, differentiation trajectories in the model need not be tree-like, as can be seen in the simulations of Fig. 3D. As *β* increases, multiple steady states can appear and disappear, and these new steady states may be averages of overlapping terminal states. The key prediction of the model is that these states will correspond to averages of terminal states with high internal similarity. For example, it is experimentally established that dendritic cells can emerge from both the myeloid lineage through macrophage-dendritic progenitors and from the lymphoid lineage through B cell-biased lymphoid progenitors (Anderson et al., 2021). This observation aligns with our model and simulations (Fig. 3D, see also Fig. S4 for simple examples of tripotent progenitors and deviations from tree structure). The gene expression profile of dendritic cells exhibits high cosine similarity to both B cells and macrophages (and, more generally, myeloid cells), whereas B cells are less similar to myeloid cells. Similarly, neutrophils may arise from distinct monocyte-neutrophil and basophil-eosinophil-neutrophil progenitors, and monocytes from distinct monocyte-neutrophil and monocyte-dendritic cell progenitors (Weinreb et al., 2020; Yampolskaya et al., 2023). These patterns are also recapitulated in our simulations and are related to the similarity structure of the cell types. Higher-level progenitor structures can be captured in the same manner by the model. For example, lymphoid and granulocyte cells have moderate cosine similarity, as do granulocytes and erythro-megakaryocytes. However, lymphoid and erythro-megakaryocytes are poorly correlated. Consequently, each of the first two pairs (lymphoid and granulocyte cells, and granulocytes and erythro-megakaryocytes) can share accessible progenitors, whereas the latter two (lymphoid and erythro-megakaryocytes) are less likely to do so. Indeed, the first two progenitors exist (lympho-myeloid primed progenitors and common myeloid progenitors), whereas the latter, to the best of our knowledge, does not. The model can thus make predictions regarding highly complex transition dynamics by using information on only the terminal cell identities.

### Evolution of new cell types

I conclude this study by considering the question of how new cell types can evolve within the regulatory network. Although in principle a new cell type may arise *de novo*, in practice it is likely to appear by the diversification of an ancestral cell type into sister cell types, a process known as genetic individuation (Arendt et al., 2016; Hobert, 2021). In this process, a cell type associated with a specific combination of TFs evolves into new and distinct cell types associated with new TF combinations.

The EnhancerNet model has unique properties that support the process of genetic individuation of cell types; I propose that these properties may have been instrumental for the evolution of distal cis-regulatory elements with dynamic chromatin in animals. These properties are (1) the ability to support multiple coexisting, highly correlated attractor states; and (2) the ability to have multiple enhancers regulating the same genes.

The importance of the first property is clear when one considers that around the time of the initial diversification of the ancestral cell types, the sister cells have highly correlated expression patterns. At this stage, they need to co-exist as distinct attractor states to be expressed in the body. Thus, in the biological context, the ability to support multiple correlated attractor cell type states is crucial. This is supported by the mechanism underlying the EnhancerNet through the inverse temperature parameter *β*, which allows cells to discriminate between highly similar expression patterns.

As a concrete example, consider the specification of neuronal identity in *C. elegans* worms. Reilly et al. investigated the expression of TFs in the mature worm nervous system (Reilly et al., 2020). Their findings showed that each of the 118 neuron classes in *C. elegans* is defined by a unique combination of homeobox TFs, with 68 TFs exhibiting variable expression across neuronal classes. Many neuronal classes share highly similar TF codes: eight classes differ from another class by only a single TF, whereas 70 classes differ from another by three or fewer TFs. The mechanism underlying Eqn 2, through regulation of the *β* parameter, successfully supports these patterns, with around a twofold increase in *β* allowing full specification of all neuronal classes from a single multipotent progenitor (see Materials and Methods).

**Fig. 4.**
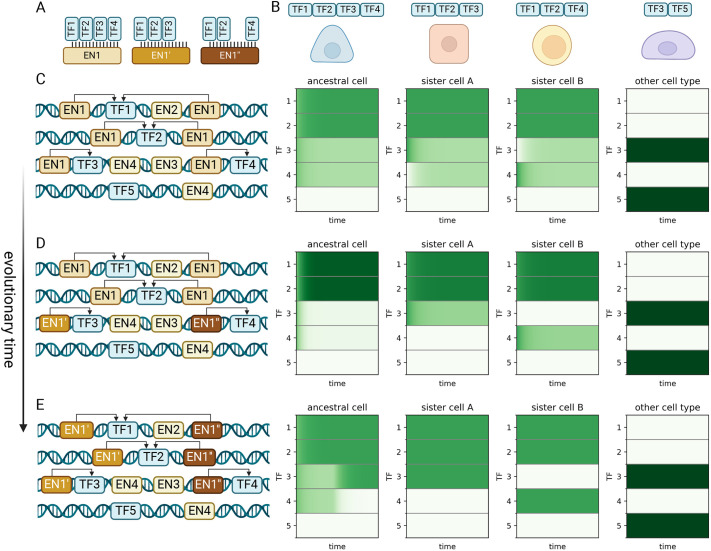
**Evolution of new cell types.** (A,B) I consider the evolution from a cell type associated with the binding pattern of TF1-4 (EN1 in A, left cell in B) to two sister cell types, each associated with the binding of TF1 and TF2 and either TF3 or TF4 (EN1′ and EN1″ in A, middle two cells in B). This must occur without interference with other cells in the regulatory network that may share TFs with these cell types. (C-E) The evolution occurs in the presence of other cell types in the gene regulatory network. (C) The original setup (left) stabilises only the ancestral cell type: see right three panels with simulations starting from the ancestral state, where TF1-TF4 are active; from sister cell A, where TF1-TF3 are active; and from sister cell B, where TF1, TF2 and TF4 are active. (D) Mutations in specific enhancers can stabilise states near all cell types. (E) Further mutation can destabilise the ancestral state. For all simulations, I used the full dynamics of Eqn 1, with the shade of green corresponding to expression strength of the TF.

The second property allows the regulatory network to generate and modify specific cell types without interfering with other cell types that may have common TFs. This relates to the modularity of encoding cell types by enhancer sequences. I illustrate this by a simple example where an ancestral cell type expressing TFs 1, 2, 3 and 4 is diversified into two cell types that express 1, 2 and 3 or 1, 2 and 4 (Fig. 4A,B). The initial attractor state is associated with an enhancer type, denoted EN1, that binds all four TFs. In our example, this enhancer is initially placed in the genome near all four TFs. It is also assumed that multiple copies, known as shadow enhancers (Hong et al., 2008; Cannavò et al., 2016; Kvon et al., 2021), of EN1 are near TF1 and TF2 (Fig. 4C). This phenomenon is widespread and well-established experimentally, and its observed behaviour is in line with model predictions (see Materials and Methods). This setup is illustrated in Fig. 4C, and corresponds to a stable attractor where TFs 1-4 are active.

Let us consider the possibility that enhancers near TF3 and TF4 evolved so that the enhancer near TF3 does not bind TF4, and the enhancer near TF4 does not bind TF3 (Fig. 4D). Now, there are three stable states: one where TF1-4 are active (with reduced activity of TF3 and TF4) and two where TF1 and TF2, and either TF3 or TF4 are active, which corresponds to the new sister cells. Over time, the shadow enhancers may evolve further, leading to the destabilisation of the original attractor state (Fig. 4E). Throughout this duration, the other cell types remain stable attractors. Thus, the EnhancerNet mechanism allows the evolution of new cell types without interfering with existing cell types.

## DISCUSSION

Here, I have derived a predictive mechanistic model for the dynamics of cell identity, based on interactions between TFs and enhancers. The model incorporated several features of the architecture of the regulatory network, i.e. that enhancers form dense autoregulatory networks with TFs and that enhancer activity is determined by its chromatin state, which is set by TF binding. These features are sufficient to derive a simple and tractable model that can be used without the need to fit unobserved parameters, and that recapitulates the known processes of cell type specification, reprogramming and differentiation.

The mechanism underlying enhancer selection in EnhancerNet is mathematically related to a recent model for memory storage and retrieval known as Modern Hopfield networks (Ramsauer et al., 2020 preprint; Krotov and Hopfield, 2021). Classical Hopfield networks are based on direct and additive interactions between components (Hopfield, 1982). Classical Hopfield networks have long been considered important conceptual models for memory storage and retrieval in the brain (Hopfield, 1982; Krotov and Hopfield, 2021), and, more recently, they have been employed in pioneering studies as conceptual and predictive models for cell fate specification, and as the basis of computational methods to study cell state dynamics (Lang et al., 2014; Fard et al., 2016; Guo and Zheng, 2017; Conforte et al., 2020; Boukacem et al., 2024). It was not clear how such a network could be implemented mechanistically in cells. Modern Hopfield networks extend Classical Hopfield networks and allow the storage of many patterns through higher-order interactions (Krotov and Hopfield, 2016, 2021). I show that their dynamics arise naturally in cells by the interactions of TFs and enhancers. The mathematical analogy between Modern Hopfield networks and enhancer-TF interactions provides a mechanism through which cells can retrieve many attractor patterns encoded by a biochemical regulatory network.

The model makes specific testable predictions for processes of reprogramming and differentiation, based on the identities of the terminal states. The model can predict reprogramming ‘recipes’ by which TFs can transition a cell from a given cell type to a target cell type. These predictions are consistent with known algorithms for this purpose and recapitulate established reprogramming recipes. The model thus provides a flexible computational framework to model reprogramming dynamics.

For differentiation, the model predicts the identity of progenitors and can predict complex differentiation hierarchies by using only the identities of the terminal cell types. Specifically, the model predicts that, in cases where a single multipotent progenitor gives rise to multiple cell types, more-restricted progenitors will preferentially appear between sets of cell types with similar expression profiles that are distinct from other cell types. The model recapitulates the complex and well-characterized differentiation hierarchy of haematopoiesis with no fitting parameters. In another well-studied system, intestinal stem cells differentiate into secretory progenitors that then give rise to specific progenitors for Paneth and goblet cells and enterochromaffin and non-enterochromaffin enteroendocrine cells (Singh et al., 2022). Singh et al. showed that the progenitors exhibit multilineage priming at both the transcriptional level, with low-level expression of cell type-specific transcripts, and at the chromatin level, with intermediate chromatin accessibility signatures at cell type-specific enhancers. The identity of these progenitors, along with their epigenetic and transcriptional profiles, aligns closely with the model predictions that are based on the similarities among these cell types.

Both the haematopoietic and intestinal systems are thus consistent with an annealing model for differentiation into multiple cell types. From a functional point of view, the annealing strategy corresponds to well-established techniques from physics and optimization to settle a system at a global minimum (Kirkpatrick et al., 1983; Van Laarhoven et al., 1987). From a biological perspective, this allows progenitor and stem cells to identify the cell types induced by signalling pathways (illustrated in Fig. 3B) and direct their differentiation towards them, avoiding convergence to metastable states. The mechanism also supports the balanced production of multiple cell fates, as demonstrated by the simulations for haematopoiesis (Fig. 3D). Annealing thus provides a solution to a key problem of the cell identity network – the need to encode multiple stable configurations (including stem and terminal configurations) while allowing transitions between these configurations, which are sensitive to upstream signalling.

Our analysis predicts that differentiation from a multipotent to a specified cell identity occurs through an increase in *β*. In our model, *β* is proportional to the ratio of global production to removal of latent chromatin modifications. A key candidate mechanism for this process is histone acetylation, a hallmark of active enhancers that plays a crucial role in transcription initiation (Creyghton et al., 2010; Kondo et al., 2014; Narita et al., 2021, 2023). The model predicts that a decrease in overall histone acetylation activity, corresponding to a reduction in *β*, will lead to loss of cell identity, whereas a decrease in the rate of histone deacetylation activity, corresponding to an increase in *β*, will promote cellular differentiation. These predictions align with well-established effects observed upon the respective inhibition of histone acetyltransferases (Ebrahimi et al., 2019; Gomez et al., 2009; Lipinski et al., 2020; Zhang et al., 2021; Narita et al., 2021; He et al., 2021) and histone deacetylases (Marks et al., 2000; Hsieh et al., 2004; Karantzali et al., 2008; Kondo et al., 2014; Li et al., 2014).

Other mechanisms, in addition to histone acetylation, are known to play a role the regulation of enhancer activity. These include the activity of chromatin remodellers such as the SWI/SNF, Mi-2/NuRD, SET1/MLL and Polycomb complexes, and involve various mechanisms, including the depletion and modification of histone proteins (Gao et al., 2009; Whyte et al., 2012; Iurlaro et al., 2021; Wolf et al., 2023; Chan et al., 2018). In addition to these, the methylation of DNA itself can play a role in regulating enhancer function (Angeloni and Bogdanovic, 2019). Some epigenetic mechanisms may show bistability through local positive-feedback regulation, resulting in digital activation patterns (Dodd et al., 2007; Angel et al., 2011; Haerter et al., 2014; Berry et al., 2017; Sneppen and Ringrose, 2019; Movilla Miangolarra et al., 2024). This property may confer robustness to cell types and protect against spontaneous reprogramming. Specifically, I considered the case where enhancer activity acts as a bifurcation parameter for a bistable switch that inhibits itself (see Materials and Methods). In such a case, a drop in enhancer activity below a critical threshold results in positive feedback that further decreases the basin of attraction for the corresponding cell type, resulting in a barrier for reprogramming.

The model can explain how new cell types can evolve without affecting pre-existing cell types encoded by the network. This applies both to evolutionary dynamics between generations of organisms and to the evolution of cells in diseases such as cancer. The model aligns with the experimentally observed robustness of shadow enhancers, which are thought to play a crucial role in the evolution of cellular diversity. The predictions of the model could be tested by synthetically engineering new cell types, through mutating shadow enhancers as prescribed by the model.

In conclusion, I propose that EnhancerNet provides a simple predictive framework for the dynamics of the gene regulatory network that control cell identity, and a basis for dissecting the complex processes of cell reprogramming, differentiation and evolution.

## MATERIALS AND METHODS

### Derivation of general EnhancerNet model

I modelled transcriptional activation by considering a process whereby transcription is initiated at enhancer *i* at rate *p*_*i*_, which results in the transcription of gene *j* at a coupling rate of *q*_*i*,*j*_. As in established thermodynamic models for gene regulation, it was assumed that there is a rate-limiting step for transcription that involves the recruitment to a specific site in the genome of transcription initiation machinery (Ackers et al., 1982; Bintu et al., 2005), which may be of high multiplicity (the effect of multiplicity in the model would be to scale overall transcription, which is not important for our conclusions). It was assumed that enhancers compete for the binding of this machinery. I denote using 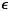
_*i*_ the energy for transcriptional initiation at enhancer *i* and assume that the transcriptional machinery is always bound to one of the enhancers. It was assumed that on the timescale of interest, which is related to changes in cell identity (days to weeks), the rate of transcription initiation is captured by equilibrium statistical mechanics. Namely, the rate of transcription initiation at enhancer *i* is proportional to the Boltzmann distribution:
(3)

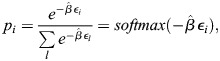
where 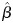 is inverse temperature. The initiation of transcription at enhancer *i* can then result in the transcription of several associated genes, and each gene can be transcribed after an interaction with one of several enhancers. The dynamics of the expression of gene *j*, denoted *x*_*j*_, is given by the sum:
(4)

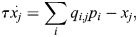
where *τ* is the timescale of gene expression changes.

Feedback in the model occurs when TFs modulate the activation energies of the enhancers. In classical models for gene regulation in prokaryotes, the binding of activator or repressor molecules directly modulates the rate of transcription initiation, corresponding to the variables 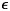
_*i*_ in our model (Bintu et al., 2005). For enhancer-mediated transcription, on the other hand, the dominant mode of enhancer activation appears to be indirect, through the modulation of enhancer chromatin (Eck et al., 2020). Binding of TFs to enhancers leads to loosening of nucleosomes and to the biochemical modification of histone proteins (Calo and Wysocka, 2013; Park et al., 2021; Hansen et al., 2022), resulting in dynamic changes in enhancer chromatin that are closely linked to enhancer activity (Heintzman et al., 2009; Creyghton et al., 2010). A dominant mode of enhancer activation appears to be the TF-mediated recruitment of histone acetyltransferases, which modify enhancer chromatin, resulting in the recruitment of transcription initiation machinery (Narita et al., 2021).

From a biophysical perspective, this can be captured by a model in which 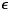
_*i*_ is set by a latent variable *m*_*i*_, which accumulates proportionally to the binding of TFs:
(5)

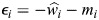

(6)

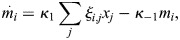
where *ξ*_*i*,*j*_ is the effective binding rate TF *j* to enhancer *i*, and where *κ*_1_, *κ*_−1_ are the associated ‘on’ and ‘off’ rates for *m*_*i*_, which may be related to the global activity of enzymes such as histone acetyltransferases and histone deacetylases. Experimentally, it was observed that the timescale of these dynamics is of the order of minutes (Weinert et al., 2018; Narita et al., 2021), and thus much faster than the typical timescale of changes in cell identity. The weight parameter 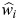 determines the energy in the absence of *m*_*i*_ or of effects on 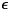
_*i*_ that are outside the cell identity feedback network.

The mechanism described in Eqn 6 is generic and may correspond to several underlying biological processes; it is, in essence, similar to modulation of receptor activation energy by methylation in bacterial chemotaxis (Tu, 2013). Taking a quasi-steady-state of *m*_*i*_ and denoting by 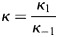, Eqn 1 is derived, with the entries of the vector 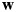 given by 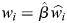 and the effective inverse temperature given by 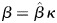.

It is also possible to consider a more general model where TF binding to enhancers is modulated at the level of the entire enhancer, e.g. by the binding of ‘pioneer’ factors that increase accessibility for other TFs. In such a case, Eqn 6 may be altered so that:
(7)

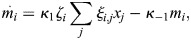
where *ζ*_*i*_ corresponds to enhancer-level gain, which effectively multiplies 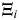.

### Equivalence of model with physical enhancers to model with enhancer types

I consider the dynamics of Eqn 1 where a subset, 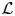, of enhancers has identical association patterns, i.e. *ξ*_*i*,*j*_=*ξ*_*k*,*j*_=*ξ*_*j*_ for all *j* and all 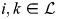. Then:
(8)

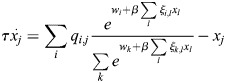

(9)

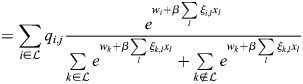

(10)

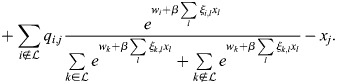
Now:
(11)

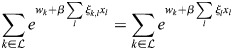

(12)

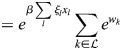

(13)

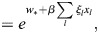
where 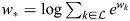. Thus,
(14)

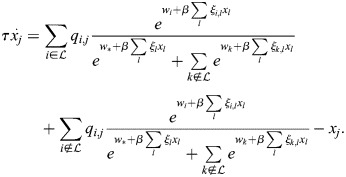
Denoting the denominator by *Z*, it was noted that:
(15)

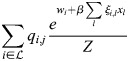

(16)

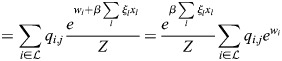

(17)

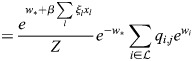

(18)

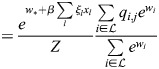

(19)

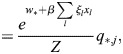
where 
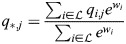
. Thus, the following may be derived:
(20)

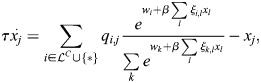
which does not include the physical enhancers of type 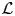, as they are replaced with a combined ‘enhancer type’ with the association constant q_*,*j*_ and weight w_*_.

### Reciprocity and autoregulation in enhancer feedback networks

Here, I will show that reciprocity:
(21)

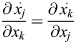
for all *k*, *j* implies that *q*_*i*,*j*_=*ξ*_*i*,*j*_ for all *i*, *j*. As the above relation is trivial for *j*=*k*, instead *j*≠*k* is taken:
(22)

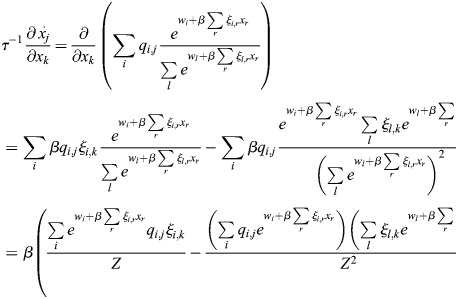
where 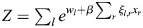.

Thus, for an arbitrary *x*, the equality 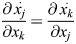 implies that *q*_*i*,*j*_=*νξ*_*i*,*j*_:
(23)

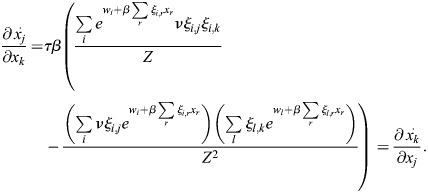
Thus, reciprocal interactions between TFs are equivalent to the autoregulation of TFs by binding to their enhancers.

### Scalar potential for transcriptional dynamics

Many derivations related to Eqn 2 appear in Ramsauer et al. (2020 preprint), including stability analysis of the fixed points, as well as storage capacity and equivalence with other machine-learning models. Here, for completeness, I will derive the important results that pertain to our paper, and refer the reader to Ramsauer et al. for more in-depth derivations that pertain to other aspects of the model. Considering the symmetric case, I will show that the dynamics are a gradient flow. A scalar potential for Eqn 2 is:
(24)

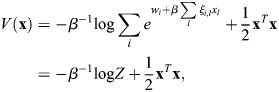
where 
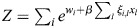
. This function is similar to the Lyapunov function of Ramsauer et al. (2020 preprint), and accounts for the bias 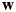. To see that the dynamics are a potential flow:
(25)

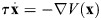
observe that
(26)

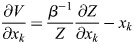

(27)

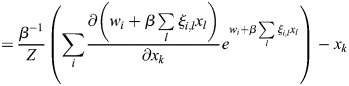

(28)

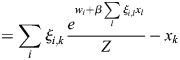

(29)

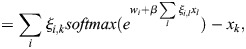
which provides the dynamics of 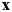.

### Rows of Q are fixed points at high *β*

Consider the row vectors of 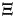 given by 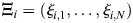. The dynamics of Eqn 2 at 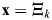 are given by:
(30)

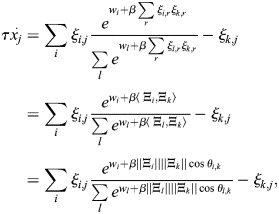
where *θ*_*i*,*j*_ determines the angle between 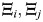 and the magnitude is defined by the Euclidean norm. It was assumed that the rows of 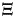 are all of magnitude unity, which is equivalent to assuming that they have comparable overall binding affinities. In this case:
(31)

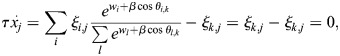
where the last equality holds for sufficiently large *β* and assuming the rows of 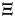 are distinct, because, in that case cos*θ*_*i*,*k*_=1 if (and only if) *i*=*k*. Thus, for a large enough *β*, the rows of 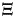 are fixed points of the dynamics. In practice, it is only important that cell types with high cosine similarity have similar overall binding affinities. Consider, for example, the case where, for pattern *k*′:
(32)

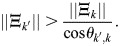
In such a case, the pattern *k* will be unstable even at very large *β*, and thus such a pattern will not be accessible. For this reason, it is expected that similar cell types will have comparable overall binding affinities. In cells, this could be achieved either through evolution of enhancer composition or by population-level negative feedback, such as the coupling of the enhancer-level gain parameter (*ζ*_*i*_) to the production of cell type *i*.

What about the case where the matrix 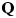 is distinct from 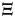
? Consider then dynamics at 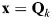:
(33)

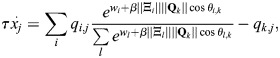
where now *θ*_*i*,*j*_ determines the angle between 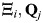. Now, in order for the pattern 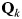 to be a fixed point at high *β*, it is required that:
(34)

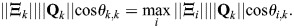
Under the assumption that the rows of 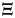 are comparable, 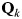 is a fixed point of the dynamics at sufficiently large *β* when there is high cosine similarity between 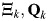 compared with the other rows of 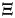.

### Averages of rows of 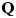 can be fixed points

Let us denote using 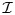 a subset of indices of enhancers of magnitude 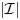 and consider the averaged vector:
(35)

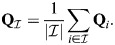
Setting 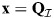 gives the dynamics:
(36)

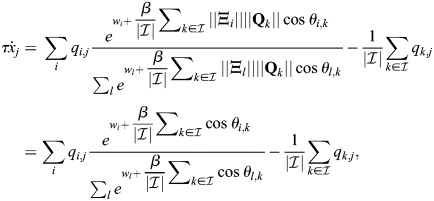
where in the last equality it was assumed that the rows of 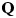 are also unity. In the case where all weights *w*_*i*_ are similar, this will be zero trivially when *β* is very small and where 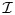 is the set of all enhancers (all *K* rows of 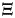), as in this case:
(37)

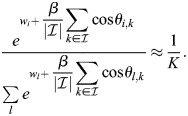
This can be generalized in a straightforward manner to the case where some of the weights of *w* are large and of comparable magnitude. Otherwise, and assuming for simplicity that 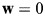, it is required that:
(38)

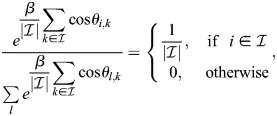
which, at large enough *β*, occurs when the averaged cosine similarity of a set element with the other elements of the set:
(39)

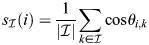
is both (1) comparable within the set, 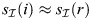 for 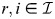, and (2) larger than outside the set, 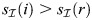 for 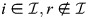. An averaged pattern of a subset will therefore be a fixed point if each pattern in the subset is similar to each of the other patterns, and distinct from patterns outside the subset. Condition (1) is specifically easy to satisfy for subsets with only two patterns; in this case, their average (a ‘bipotent progenitor’) will be a fixed point when they have large cosine similarity and are distinct from other patterns.

### Global stability of averages depends on *β*

To probe the global stability of averages of rows of 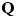, the scalar potential *V* can be used. The dynamics proceed from high *V* to low *V* so it is expected that states with higher *V* are less likely to be stable than states with lower *V*. As the potential function is only defined for the symmetric case, the states are defined as averages of the rows of 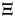, which are analogously defined as 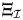, where 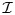 is as before a set of indices:
(40)

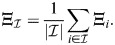
Setting 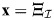, the following may be evaluated:
(41)

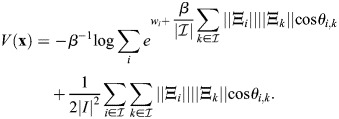
Again, taking the magnitude of the rows of 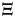 to be unity and 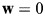 (equivalent to enhancers with similar binding strengths and weights) gives:
(42)

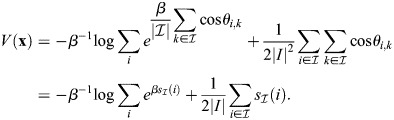
The energy of averaged patterns thus depends only on their cosine similarity relative to all patterns (first term) and internal cosine similarity (second term). As an illustrative case, let us assume that 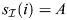 for 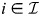 and 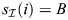 for 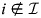 [considering that, in general, *A*, *B* are between (−1, 1)]. Then:
(43)

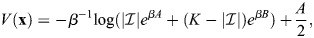
where *K* is the overall number of patterns. The case of comparable *A*≈*B*, corresponding to random subsets, gives:
(44)

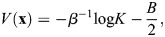
which for large *β* would correspond to 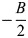 and will thus have high energy when *B* is low. On the other hand, in the case where *A*≫*B*, as a rough approximation:
(45)

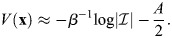
When *β* is very large, the size of the subset |*I*| does not contribute to *V* and thus it is minimized when *A* is maximized, i.e. at single patterns with *A*=1 (where 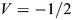). At intermediate *β*, however, larger subsets may have lower energy. For two subsets where 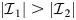 and *A*_1_<*A*_2_, the larger set will have lower energy when:
(46)

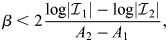
which occurs for a larger range when the difference between the set magnitudes is larger and the difference between their average cosine similarity is smaller. Specifically, the differentiation from a bipotent progenitor 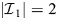 to a terminal identity 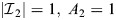 occurs at a critical *β*:
(47)

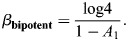
Thus, an annealing strategy where *β* is decreased and then slowly increased results in a transition from large (global) averages, which correspond to multipotent progenitors, to a set of ever more restricted progenitors, similar to the ‘Waddingtonian’ dynamics.

### Temperature transitions for cell type evolution

In this section, I will estimate the temperature *β* required to specify a population of related cell types. These cell types may share activity patterns across many of their TFs and differ in some smaller regions. As an example that will be discussed later, neurons may have similar TF activity patterns across many TFs, yet small differences in TF activity can fine-tune the identity of specific neurons.

I will specifically consider *K* patterns associated with closely related cell types and *K*′ patterns associated with other cell types. There are *N* bits that differ between the related cell types, of which a fraction *η* are active (drawn at random), *N*″ bits that are inactive and *N*′ bits that are active in all other cell types. As patterns have magnitude unity, the activity level of active bits was set to 
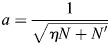
. It was assumed that unrelated patterns have *ηN*+*N*′ active TFs drawn at random. The average inner product of the related cell types is given by:
(48)

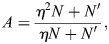
while the average inner product between random cell types is given by:
(49)

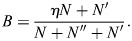
Let 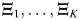 denote each of the closely related patterns, and 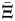 their average. The energy of each pattern is given by:
(50)

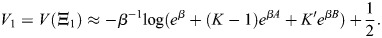
It was assumed that the patterns are sufficiently well separated (*A*≫*B*) and that *β* is sufficiently large, such that:
(51)

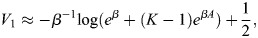
while the energy of the averaged pattern is:
(52)

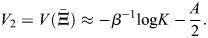
In general, as *β* increases, it would be expected to see first a transition from the global average to the local average of the two patterns, which occurs when *V*_1_ drops below *V*_2_. This transition occurs at a critical value of *β* that satisfies (assuming *K*≫1):
(53)

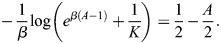
When *β* is small then 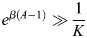 and the l.h.s. is equal to 1−*A* and thus larger than the r.h.s. When 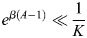 the l.h.s. is negative and thus smaller than the r.h.s. Thus, the critical *β* occurs around:
(54)

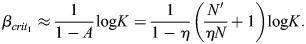
I will now consider the case where a new cell type 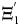 evolves from 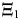. I will assume, without loss of generally, that the evolved pattern 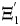 differs from the existing pattern 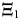 by two bits, i.e. *ξ*_1,1_=*a*, *ξ*′_1,1_=0, *ξ*_1,2_=0, *ξ*′_1,2_=*a*. The energy of the new patterns is given by:
(55)

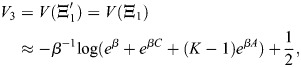
with *C* corresponding to the inner product of the new cell types:
(56)

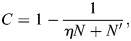
whereas the energy of the averaged pattern is:
(57)

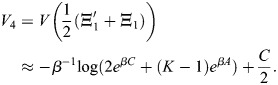
For the second transition, the case of interest is 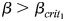, where the contributions of the (*K*−1)*e*^*βB*^ terms to the energy levels are negligible. In this case, Eqn 47 can be used to give:
(58)

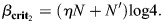
Using Eqns 58 and 54, a ratio *χ* can be derived that captures the relative increase in *β* required to evolve and stabilize a new cell type from an existing population of cells:
(59)

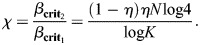
The gain *χ* thus depends only on the TFs that are variable between the cells and, among these TFs, scales with the variance of the active TFs. In the case of neuronal identity specification in *C. elegans*, the expression code is sparse, with *μ*=0.1 among variable TFs, with the other parameters being *K*=118 neuron classes and *N*=68 TFs exhibiting variable expression across neuronal classes. For this system, this gives:
(60)

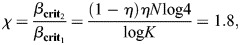
implying a further 80% increase in *β* required from the initial destabilization of a multipotent progenitor to the complete specification of all cell types.

### Local positive feedback as a barrier for reprogramming

Consider a modification to the EnhancerNet model where the energy for transcriptional activation is dependent on both the original modification *m*_*i*_ and a new modification *u*_*i*_:
(61)

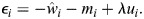
Here, *λ* is a scalar and *u*_*i*_ is autocatalytic and inhibited by enhancer activation. I propose the following dynamics for *u*_*i*_:
(62)

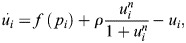
where *n*≫1 and *f*(*p*_*i*_) is a decreasing function of enhancer activity *p*_*i*_. Eqn 62 can exhibit either bistability or hysteresis, depending on the value of *ρ*. It was assumed that initially *u*_*i*_=0 and *p*_*i*_ is sufficiently large (as in the multipotent state), maintaining stability at *u*_*i*_=0. As cells differentiate, the activity of specific enhancers decreases while others increase. When the activity of an enhancer drops below a critical threshold, *p*_*crit*_, Eqn 62 undergoes a bifurcation, causing *u*_*i*_ to increase to a much larger value (*u*_*i*_≫1). This increase may persist due to a transition to a new fixed point when the dynamics are bistable. Consequently, the energy required to initiate transcription from the enhancer increases, making reprogramming to the associated cell type (whether induced by signalling, overexpression of specific transcription factors, or noise) less likely. This mechanism effectively creates a barrier to reprogramming.

### Direct reprogramming through the overexpression of TFs

Consider a transcription factor *x*_*j*_ that is constitutively overexpressed to an extent *δ*_*j*_. This constitutive expression alters energy levels 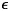
_*i*_ without feedback. This therefore takes into account only the effect of the additional TF production on the energy levels of the enhancers, without altering the coordinates of the stable fixed points. *V*′ denotes the potential under the perturbed dynamics. Evaluating *V*′ at pattern *k* yields, at high *β*:
(63)

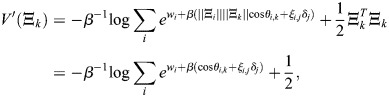
where it was assumed that 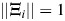 for all *i*. Assuming that unperturbed pattern *k* is stable at a given *β*, the summand for *δ*_*j*_=0 takes its maximum at *i*=*k*, where:
(64)

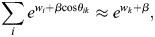
thus:
(65)

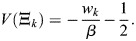
Under the assumption that the perturbation magnitude is relatively small (and, generally, for patterns that are stable after the perturbation):
(66)

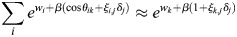
and thus:
(67)

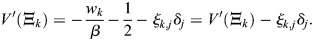
Note that Eqn 67 readily generalizes to a perturbation in several TFs 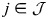:
(68)

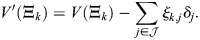
If the goal is to steer the dynamics toward a pattern *k*, then the TFs 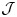 and their overexpression need to be chosen such that 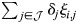 is sufficiently larger for *i*=*k* than for all other patterns *i*≠*k*. That is, the ideal expression for a TF *j* is such that *ξ*_*i*,*j*_ is large for *i*=*k* (amplifying *δ*_*j*_) and zero for *i*≠*k*.

### Preprocessing and simulation

The matrix 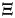 was initialized from transcript counts extracted from murine cells and averaged over cell type. For the Tabula Muris dataset (Schaum et al., 2018), I used all available data across organs and averaged over cell type. For haematopoiesis, I used the Haemopedia dataset (Choi et al., 2019) and averaged all cells that belong to the same terminal lineage. The data were then log-transformed (using a log1+*x* transformation) and filtered for TFs with mean and std expression larger than log4, as well as for Tabula Muris the TFs that participate in the tested reprogramming pathways (a full list of cell types and TFs used can be found in Tables S1-S4). Although, in principle, a log transformation is not needed for our model, which does not assume log-transformed values of *x*, it has the statistical advantage of reducing variance in the rows of 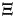 and thus reducing sensitivity to TF choice. Finally, the rows of 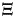 were normalized to unity. Simulations were performed using Python, taking *τ*=1, and the (terminal) *β* was chosen so that all patterns were stable.

### Simulation of reprogramming

To simulate forced TF expression during reprogramming, the following dynamics may be used:
(69)

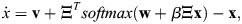
where 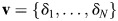 is a vector where *δ*_*i*_ corresponds to the degree of activation of TF*i*. In general, it was assumed that *δ*_*i*_=1 when the TF was in the reprogramming pathway and *δ*_*i*_=0 when it was not. However, for a few genes, setting *δ*_*i*_ as different from unity was necessary to achieve the correct reprogramming (setting *δ*_*i*_=1 achieves reprogramming to a closely related cell type). The full list of reprogramming pathways and relevant *δ* values can be found in Table S5.

### Simulation of balanced differentiation

Haematopoiesis was simulated by using annealing from *β*=0 to *β*=50 over 50 time units. The initial state corresponded to the averaged value of all terminal states. Simulations were performed by adding additive white noise:
(70)

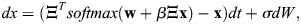
with *σ* set at 0.01. To simulate balanced differentiation in haematopoiesis, I used the following feedback procedure that aims to imitate *in vivo* feedback on differentiation, where there is negative feedback on the production of each cell type by signalling. 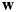 was initialized as a zero vector. Then, running from *k*=0 to *k*=*k*_*max*_, we performed differentiation by annealing. If the outcome terminal cell corresponded to row *i* of 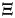, I adjusted *w*_*i*_=*w*_*i*_−0.5(1−*k*/*k*_*max*_) and then mean-adjusted *w* to zero. This resulted in a signalling profile, *w*, that produced all cell fates from the initial progenitor population.

## Supplementary Material



10.1242/develop.202997_sup1 Supplementary information
