# Supplementary material for: EnhancerNet: a predictive model of cell identity dynamics through enhancer selection
Source: Development. 2024 Oct 9;151(19):dev202997. doi: 10.1242/dev.202997 (PMC11488642; doi:10.1242/dev.202997)
Supplement: Supplementary information [file develop-151-202997-s1.pdf]

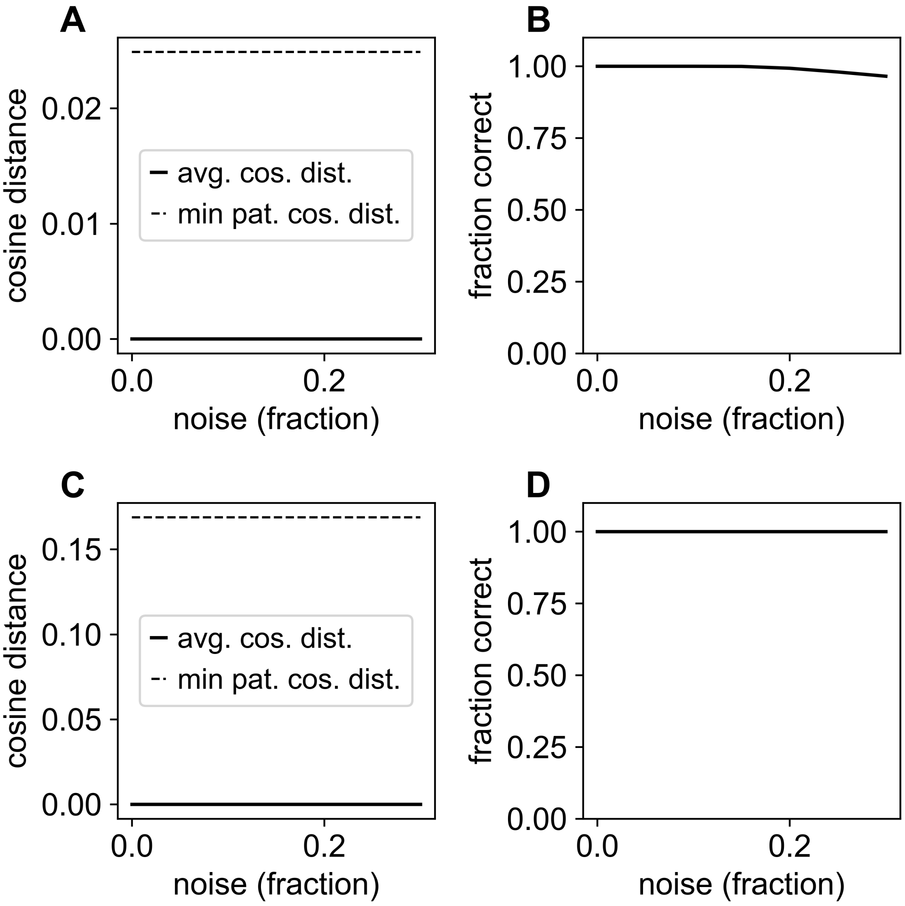

Fig. S1. Robustness of fixed points to asymmetry in enhancer-TF interactions. Throughout the manuscript, we assume symmetry in enhancer-TF interactions, captured mathematically by $\mathbf{Q} = \Xi$. However, in practice, it is likely that the matrices are correlated but not perfectly identical, that is, there is some asymmetry in the dynamics. To test for the effect of this asymmetry, we added noise to the entries of Ξ by multiplying each entry by a scalar drawn from a normal distribution with mean 1 and standard deviation ranging from 0 to 0.3 (entries were capped at a minimum of 0) while fixing the $\mathbf{Q}$ matrix. We used the Ξ matrix based on the Tabula Muris data (A,B) or a matrix initialized with uniformly drawn entries of the same dimensions (C,D). As in Figure 2, the dynamics were initialized from each of the rows of $\mathbf{Q}$. Even at very high noise magnitudes, the dynamics always settle at a cell type specified by the $\mathbf{Q}$ matrix (A,C, solid line denoting average minimal cosine distance of final state from rows of $\mathbf{Q}$, dashed line minimal cosine distance between rows of $\mathbf{Q}$). The only effect of noising is to destroy specific attractors of very similar cell types, such as T/NK cells, by destroying their distinguishing features (B,D, line capturing average fraction of final states that are identical to the initial state).

Development • Supplementary information

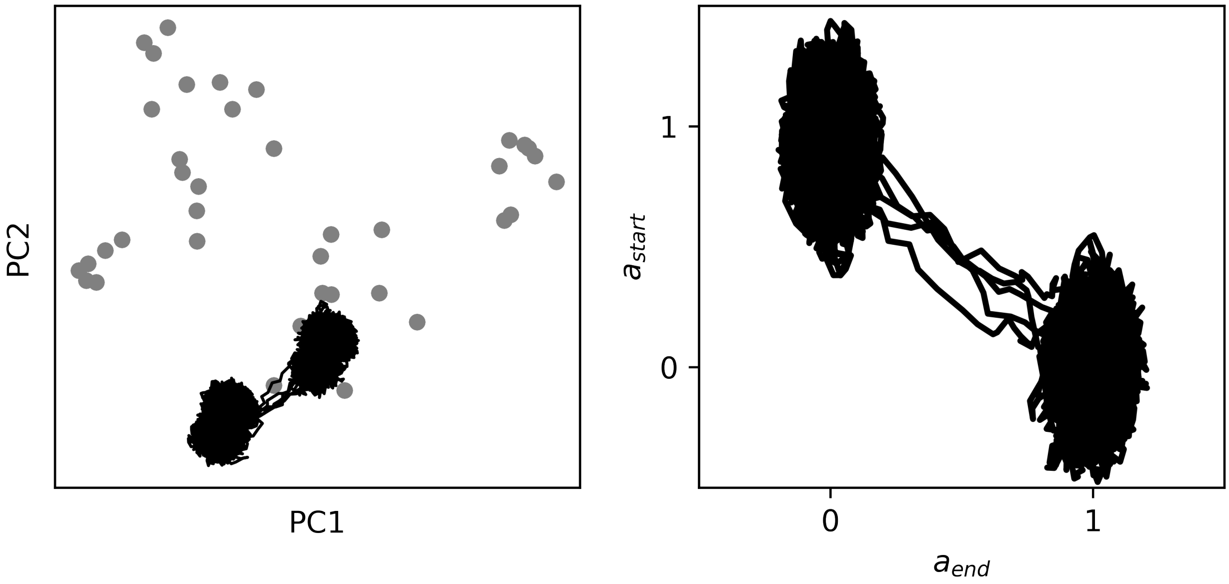

Fig. S2. Stochastic reprogramming dynamics. Here we considered the stochastic dynamics during reprogramming by using the stochastic version of the EnhancerNet model (Methods), setting $\sigma = 0.02$, and a modified reprogramming weight of $\delta = 0.85$ for the TF *Ascl1*. The initial condition is set to the fibroblast state. Here, the transition from fibroblast to neuron is not deterministic, but occurs stochastically as a memoryless barrier crossing. The left panel displays the dynamics after PCA transformation on all the cell types, while the right panel displays the dynamics transformed by an orthogonal projection of the TF profiles of the tabula muris cell types, as in Lang et al. 2014; Pusuluri et al. 2017. Here a_{start} corresponds to projection of the dynamics on the fibroblast cell fate and a_{end} corresponds to the projection on the neuron state.

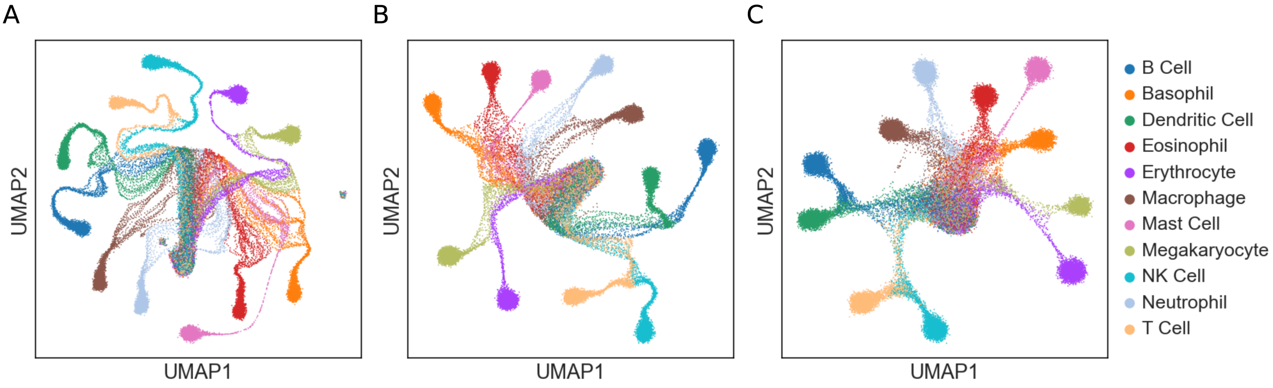

Fig. S3. Blood differentiation with different noise magnitudes. Differentiation in hematopoiesis, as in Fig. 3D, was simulated by taking (A) $\sigma = 0.003$, (B) $\sigma = 0.01$, (C) $\sigma = 0.03$, and then plotted as in Fig. 3D.

Development • Supplementary information

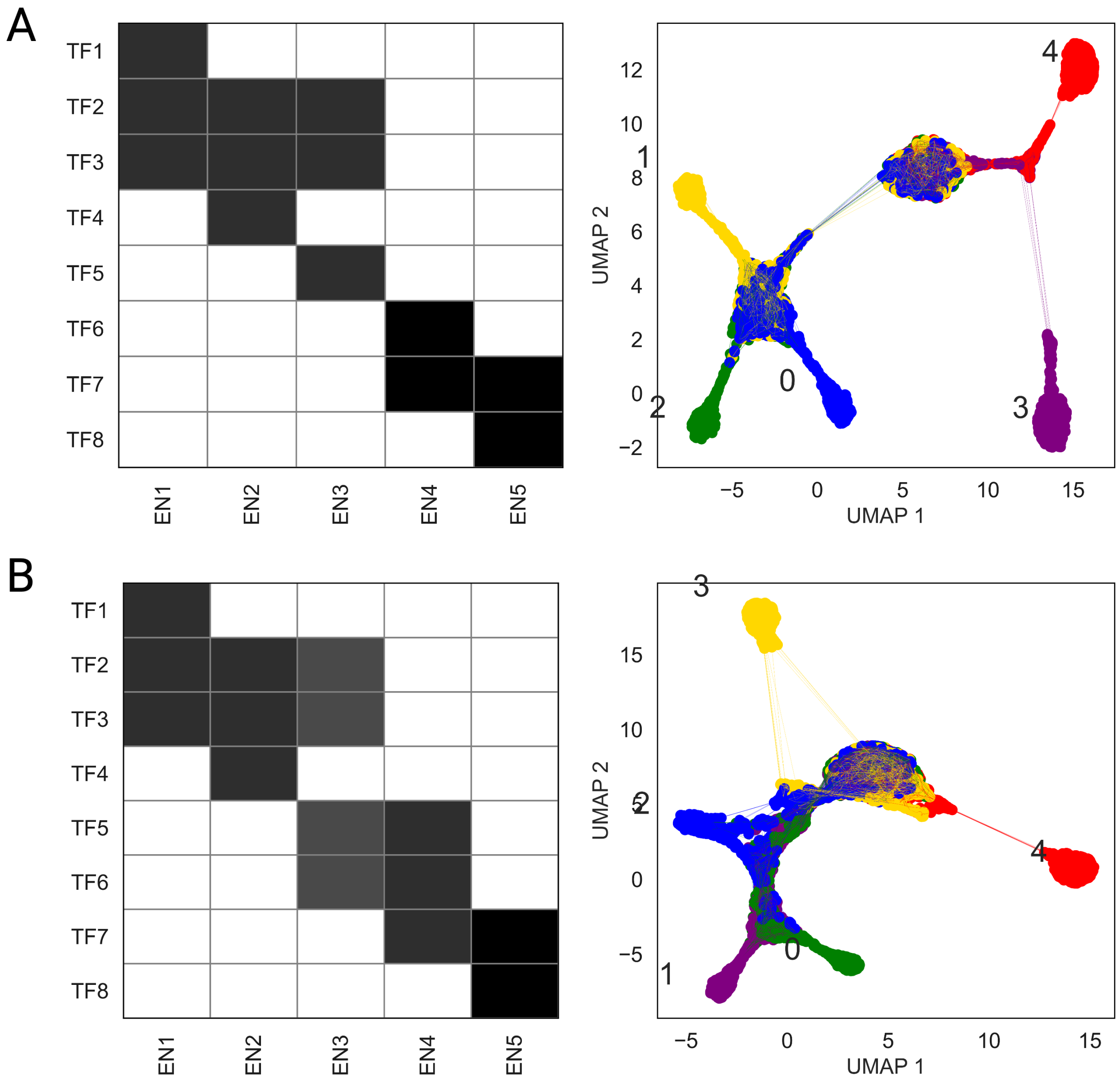

Fig. S4. Tripotent and bipotent progenitors. (A) Differentiation by annealing, where the first three cell types and the last two cell types have highly correlated expression patterns. Differentiation proceeds through a tripotent progenitor for the first set of cell types, and a bipotent progenitor for the latter. (B) Differentiation by annealing, where there is a complex correlation structure, can result in deviation from tree-like structure with multiple paths for differentiation to the terminal cell types. In all simulations, we used $\sigma = 0.05$ and a maximum annealing inverse temperature of $\beta = 12$.

Development • Supplementary information

Table S1. List of transcription factors used in Fig. 2

Gene Name	Gene Name	Gene Name
Zeb2	Hey1	Plscr2
Csrnp1	Trps1	Egr2
Tal1	Zfp536	Sp110
Fosl2	Zfhx3	Ehf
Tbx3	Sox5	Tshz2
Tbx5	Myc	Mafb
Nr1d1	Foxo1	Etv2
Epas1	Klf15	Foxa3
Neurog3	Lmo2	Gata3
Egr1	Sox9	Mnx1
Akna	Cxxc5	Zeb1
Hnf4a	Gata4	Isl1
Neurog2	Maf	Klf4
Foxa1	Hmgn3	Myt1l
Meis1	Creb3l2	Cebpb
Nfia	Satb1	Gata6
Sox10	Irf8	Pou3f2
Meis3	Pparg	Fli1
Fos	Mafa	Aebp1
Ebf1	Foxa2	Klf2
Elf3	Hes1	Tcf4
Ets1	Plagl1	Tsc22d1
Nfil3	Ascl1	Runx1
Nr2f2	Irf5	Nr1h3
Mef2c	Zbtb16	Zfp9
Nfix	Sp100	Etv1
Myod1	Rora	Plscr1
Tcf7l1	Nr4a2	Tfcp2l1
Zbtb20	Meis2	Klf5
Trp63	Zfp612	Vdr
Cebpa	Pdx1	Nr4a1
Atf3	Peg3	Irf7
Fosb	Bhlhe40	Tgif1
Creb3l1	Nfib	Irf6
Arid5a	Olig2	Cebpd
Lhx3		

Table S2. List of cell types used in Fig. 2

Cell Type	Cell Type	Cell Type
Bergmann glial cell	Brush cell of epithelium proper of large intestine	Kupffer cell
Astrocyte	Basal cell	Basophil
Bladder cell	Bladder urothelial cell	Brain pericyte
Cardiac muscle cell	Ciliated columnar cell of tracheobronchial tree	Endocardial cell
Endothelial cell of hepatic sinusoid	Enterocyte of epithelium of large intestine	Enteroendocrine cell
Epidermal cell	Epithelial cell of lung	Epithelial cell of proximal tubule
Erythrocyte	Fibroblast	Hepatocyte
B cell	T cell	Natural killer cell
Keratinocyte	Kidney collecting duct epithelial cell	Large intestine goblet cell
Luminal epithelial cell of mammary gland	Lung endothelial cell	Macrophage
Microglial cell	Monocyte	Myofibroblast cell
Neuron	Oligodendrocyte	Pancreatic a cell
Pancreatic d cell	Pancreatic pp cell	Pancreatic acinar cell
Pancreatic ductal cell	Pancreatic stellate cell	Skeletal muscle satellite cell
Smooth muscle cell	Stromal cell	Type b pancreatic cell

Table S3. List of transcription factors used in Fig. 3

Gene Name	Gene Name	Gene Name
Zfp385a	Tcf7	Bcl11a
Mxd1	Tbx21	Spi1
Klf4	Fosb	Mef2c
Spib	Pou2f2	Gata2
Rxra	Ikzf3	E2f2
Myb	Fos	Irf4
Bcl6	Hes1	Nr4a1
Satb1	Ikzf2	Atf3
Gfi1b	Creb3l1	Tal1
Fosl2	Gfi1	Irf5
Bhlhe40	Gata1	Ets1
Pou2af1	Ltf	Lyl1
Jdp2	Cebpa	Rara
Prdm1	Runx2	Hlx
Bach2	Erg	Meis3
Irf8	E2f8	Cxxc5
Zfpm1	Cebpe	Klf2
Maf	Cebpb	Nfil3
Nfe2	Zfp516	Hmgn3
Cebpd		

Table S4. List of cell types used in Fig. 3

Cell Type
B cell
Basophil
Dendritic cell
Eosinophil
Erythrocyte
Macrophage
Mast cell
Megakaryocyte
NK cell
Neutrophil
T cell

Table S5. Reprogramming recipes

	source cell	target cell	factors	weights
0	fibroblast	neuron	Ascl1,Lhx3,Mnx1,Isl1,Neurog2,Pou3f2,Myt1l	1,1,1,1,1,1,1
1	fibroblast	neuron	Ascl1,Pou3f2,Myt1l	1,1,1
2	fibroblast	neuron	Ascl1	1
3	fibroblast	oligodendrocyte	Sox10,Zfp536,Olig2	1,1,1
4	fibroblast	lung endothelial cell	Foxo1,Etv2,Klf2,Tal1	1,1,1,1
5	fibroblast	type B pancreatic cell	Neurog3,Pdx1,Mafa	1,5,1
6	fibroblast	hepatocyte	Foxa1,Foxa2,Foxa3,Hnf4a	1,1,1,1
7	fibroblast	cardiac muscle cell	Gata4,Mef2c,Tbx5	1,1,1
8	fibroblast	skeletal muscle satellite cell	Myod1	1
9	fibroblast	epidermal cell	Trp63,Klf4	1,1
10	B cell	monocyte	Cebpa,Cebpb	0.5,2
11	pancreatic ductal cell	type B pancreatic cell	Pdx1	5

Development • Supplementary information
