# Supplementary material for: EnhancerNet: a predictive model of cell identity dynamics through enhancer selection
Source: Development. 2024 Oct 9;151(19):dev202997. doi: 10.1242/dev.202997 (PMC11488642; doi:10.1242/dev.202997)
Supplement: Supplementary file 1 [file develop-151-202997_review_history.pdf]

# EnhancerNet: a predictive model of cell identity dynamics through enhancer selection

Omer Karin
DOI: 10.1242/dev.202997

**Editor**: Paul Francois

**Review timeline**

| | |
|---|---|
| Original submission: | 25 April 2024 |
| Editorial decision: | 26 June 2024 |
| First revision received: | 5 August 2024 |
| Editorial decision: | 3 September 2024 |
| Second revision received: | 5 September 2024 |
| Accepted: | 6 September 2024 |


---

**Original submission**

First decision letter

MS ID#: DEVELOP/2024/202997

MS TITLE: A model for enhancer selection recapitulates the dynamics of cell type acquisition

AUTHORS: Omer Karin

I have now received all the referees' reports on the above manuscript, and have reached a decision. The referees' comments are appended below, or you can access them online: please go to BenchPress and click on the 'Manuscripts with Decisions' queue in the Author Area.

As you will see, the referees express considerable interest in your work, but have some significant criticisms and recommend a substantial revision of your manuscript before we can consider publication. If you are able to revise the manuscript along the lines suggested, which may involve further experiments, I will be happy receive a revised version of the manuscript. Your revised paper will be re-reviewed by one or more of the original referees, and acceptance of your manuscript will depend on your addressing satisfactorily the reviewers' major concerns. Please also note that Development will normally permit only one round of major revision. If it would be helpful, you are welcome to contact us to discuss your revision in greater detail. Please send us a point-by-point response indicating your plans for addressing the referees' comments, and we will look over this and provide further guidance.

Please attend to all of the reviewers' comments and ensure that you clearly highlight all changes made in the revised manuscript. Please avoid using 'Tracked changes' in Word files as these are lost in PDF conversion. I should be grateful if you would also provide a point-by-point response detailing how you have dealt with the points raised by the reviewers in the 'Response to Reviewers' box. If you do not agree with any of their criticisms or suggestions please explain clearly why this is so.

Reviewer 1

*Advance summary and potential significance to field*

© 2024. Published by The Company of Biologists under the terms of the Creative Commons Attribution License (https://creativecommons.org/licenses/by/4.0/).

In this manuscript, the author proposes a new framework (EnhancerNet) for understanding how cell identity is enforced within networks of enhancers and transcription factors. In the author's proposal, transcriptional machinery binds to enhancers, which then control gene expression. Expression levels then feedback to influence enhancer binding. The resulting network can then define multiple stable fixed points corresponding to different cell types. The most novel aspect of the framework is proposing that modulation of a temperature-like variable within the enhancer binding rate can alter the "energy" landscape and control the nature of the stable fixed points. As the temperature is "cooled" the system relaxes back into specific steady-states in a way which recapitulates known developmental dynamics in, for example, the hematopoietic system. A proposal of the evolution of new cell types is also proposed within the formalism.

*Comments for the author*

In general, I liked the proposed framework and it has considerable strengths encapsulating known dynamics in a simple way, while making interesting predictions in the pathways through which differentiation proceeds. I am therefore, in principle, very supportive of publication. However, as it stands much of the paper is very tough to read. It gets somewhat simpler towards the end, but the reality is that >90% of readers will not get through the first results section. So, a key objective that the author must satisfy is in simplifying the presentation. At the moment, the text is very dense, with only readers conversant to a high level in both experimental developmental biology and statistical physics having any chance of grasping it. The overlap of these two communities is (sadly) a fairly small set.

Another issue in the initial results section is in the numerous simplifications and assumptions that are made. At present, it is mostly unclear whether these simplifications are simply calculational conveniences necessary to be able to make analytic progress or whether these simplifications sacrifice real biology properties. Such assumptions include: the transcriptional machinery always being bound to exactly one enhancer only; assumption of Boltzmann weights when the real system will be way out of equilibrium; quasi-steady-state approximations around Eq. 5; assumption of Q=Xi when in reality the entries are only correlated; assumption of large beta (compared to what?). Another assumption in the Methods (needed to show that the rows of Q are fixed points) is that the rows are all of magnitude unity: how strong is that assumption? Later on in the section on direct reprogramming, w is additionally taken to be zero "for simplicity". Again, how important is that assumption?

For the hematopoiesis simulations, the author needs to specify more clearly how this was actually implemented. Does the homogeneous initial population mean that the initial conditions are the same for all cells or that the initial conditions homogeneously explored the parameter space? In addition, noise was added to the dynamics: how important is this noise for the generation of the correct lineages? What is the size of the noise and is that biologically realistic? How sensitive are the results (particularly the proportions) to these fluctuations?
Also I do not understand the biological rationale for the negative feedback routine used to tune the weight vectors.

It would also be helpful if the author could comment on other mechanisms for establishing stable cell type expression states. In the EnhancerNet scheme this results from fixed points of a set of TF/enhancer differential equations. But there are other possibilities where, for example, the chromatin itself locally specifies memory of the transcriptional state (through independent feedback involving Polycomb or heterochromatin or DNA methylation). How would that interface with the author's scheme?

Reviewer 2

*Advance summary and potential significance to field*

In his manuscript "A model for enhancer selection recapitulates the dynamics of cell type acquisition", Karin proposes that the essential dynamics of cell fate networks can be mathematically described using "Modern Hopfield Networks". He also proposes a mechanistic explanation for these dynamics based on enhancers and TF interactions. The work is theoretically

© 2024. Published by The Company of Biologists under the terms of the Creative Commons Attribution License (https://creativecommons.org/licenses/by/4.0/).

sophisticated and quite compelling. It shows that many aspects of cell fate can be understood using new ideas developed on attractor neural networks, and suggests a framework for analyzing new data. I think the paper is very good and ideally suited for a journal like Development.

*Comments for the author*

In his manuscript “A model for enhancer selection recapitulates the dynamics of cell type acquisition”, Karin proposes that the essential dynamcis of cell fate networks can be mathematically described using “Modern Hopfield Networks”. He also proposes a mechanistic explanation for these dynamics based on enhancers and TF interactions. The work is theoretically sophisticated and quite compelling. I think the paper is very good and ideally suited for a journal like *Development*. My main comments regard: connection to earlier literature, minor clarifications and extensions, and numerically testing how robust the construction is to introducing asymmetry in the TF-enhancer interactions.

The theoretical starting point for this work is the idea that cell fates can be viewed as attractors of a dynamical system. It is builds upon an earlier works, that the author is likely not aware of, that Hopfield-inspired attractor networks offer a compelling model of cell fate (see discussion of this in this review Teschendorff et al. *Nature Reviews Genetics* 22.7 (2021): 459-476.; Lang et al. *PLoS computational biology* 10.8 (2014): e1003734.; Pusuluri et al. *Physical Biology* 15.1 (2017): 016001.; Smart et. al. *Cell Reports Physical Science* 4.2 (2023). Yampolskaya, Maria"scTOP: physics-inspired order parameters for cellular identification and visualization." *Development* 150.21 (2023): dev201873.). The authors independently arrive at a similar model, but with three very interesting and important extensions. First, instead of using ordinary Hopfield networks they extend this construction to Modern Hopfield networks. Second, they give a mechanistic interpretation of these models in terms of enhancer-TF interactions. Third, they argue that the spurious attractors in these networks can be naturally interpreted as progenitor cell types. It would be nice if the authors could connect the current results with this earlier literature and discuss the implications of these differences for predictions.

I found the reprogramming/differentiation experiments very interesting. I would like to know something about the trajectories that are found and how the energy (Lyapunov function) differs between reprogramming and differentiation. It was found in the earlier works above that the energy for reprogramming passed through high energy states whereas differentiation does not. Is this also the case here?

I found the discussion of annealing temperature interesting. It is a property of all Hopfield like models that the low energy ``spurious attractor” are always mixtures of existing cell types, and more correlated they are, the more likely the spurious attractors (see discussion in Amit’s book *Modeling Brain Function* for nice summary of the literature). However, in earlier works such as Lang *et al*. it was argued that such attractors are likely not biologically meaningful but instead correspond to *in vitro* objects such as partially reprogrammed cells. I found the proposal here that these spurious attractors may correspond to progenitors quite interesting. The clustering in Figure 3c essentially builds a tree between closely related cells. However, recent lineage tracing experiments (Weinreb et al. *Science* 367.6479 (2020)) indicate that the picture might be more complicated, including progenitors that can give rise to three or more cell types. It was unclear if the model captures this more refined information. Also, in other lineages, I suspect that the model may predict progenitors between all closely related cell types? Are there scenarios where there is high cosine-similarity but no progenitor state? How do you reconcile this with biological experiments if there is not?

For the evolution question, I think the Modern Hopfield networks are much more stable than traditional Hopfield networks because of the exponential storage capacity. However, it is unclear to me if this evolution also requires temperature (beta) to be extremely small. It would be nice to understand how the ability to evolve depends on the choice of temperature and the loading of these networks?

Finally, my intuition is that the authors constructions are actually robust to adding a significant amount of asymmetry to the model. One easy way to test this is to numerically just add noise so that $Q=Xi +Noise$ for different levels of noise and see if the attractors are still robust. This

© 2024. Published by The Company of Biologists under the terms of the Creative Commons Attribution License (https://creativecommons.org/licenses/by/4.0/).

would show that one just needs approximate symmetry not exact symmetry, making the model more biophysically plausible.

---

**First revision**

Author response to reviewers' comments

**Reviewer 1.**
In general, I liked the proposed framework and it has considerable strengths, encapsulating known dynamics in a simple way, while making interesting predictions in the pathways through which differentiation proceeds. I am therefore, in principle, very supportive of publication.

Thank you for this strong endorsement of the manuscript.

However, as it stands much of the paper is very tough to read. It gets somewhat simpler towards the end, but the reality is that >90% of readers will not get through the first results section. So, a key objective that the author must satisfy is in simplifying the presentation. At the moment, the text is very dense, with only readers conversant to a high level in both experimental developmental biology and statistical physics having any chance of grasping it. The overlap of these two communities is (sadly) a fairly small set.

I have extensively revised the manuscript, with particular focus on the Results section, to improve its clarity and accessibility for *Development* readers. The revisions include partitioning the more technical aspects of the manuscript, including model derivation, into the Methods section, while expanding explanations throughout the text to provide greater intuition. I have added more biological context and examples to illustrate key concepts, simplified complex terminology where possible, and provided intuitive definitions where specialized terms are necessary.

Another issue in the initial results section is in the numerous simplifications and assumptions that are made. At present, it is mostly unclear whether these simplifications are simply calculational conveniences necessary to be able to make analytic progress or whether these simplifications sacrifice real biology properties. Such assumptions include: the transcriptional machinery always being bound to exactly one enhancer only; assumption of Boltzmann weights when the real system will be way out of equilibrium; quasi-steady-state approximations around Eq. 5; assumption of Q=Xi when in reality the entries are only correlated; assumption of large beta (compared to what?). Another assumption in the Methods (needed to show that the rows of Q are fixed points) is that the rows are all of magnitude unity: how strong is that assumption? Later on in the section on direct reprogramming, w is additionally taken to be zero "for simplicity". Again, how important is that assumption?

I now clarify in the manuscript the precise role and importance of the simplifications and assumptions that I am using.
*Transcriptional machinery:* While multiple instances of the transcriptional machinery may exist, each binding some enhancer at any given time, we do not need to model this explicitly, as the effect of multiple instances would scale overall transcription, which is not important for our analysis.
*Boltzmann weights:* The model assumes that competition between enhancers for binding to transcriptional machinery can be captured by (or at least approximated by) the Boltzmann distribution. Similar models are common in gene regulation studies and other cellular processes. In our case, this assumption needs only hold on the relevant timescale of cell identity changes—a point clarified in the revisions. While more complex EnhancerNet dynamics are possible, I believe that the current model, based on equilibrium (or quasi-equilibrium) dynamics, sufficiently captures the central hallmarks of cell identity regulation, and can be extended in future research.
*Quasi-steady-state approximation:* The quasi-steady-state approximation for the dynamics of *m* is

© 2024. Published by The Company of Biologists under the terms of the Creative Commons Attribution License (https://creativecommons.org/licenses/by/4.0/).

inconsequential for our analysis and is only needed to simplify the model expression, as this work focused on steady-state and on slow changes in cell identity. There is also experimental evidence that the main mechanism implicated, namely enhancer histone acetylation, occurs with rapid turnover of minutes, compared with processes of differentiation that take days-weeks (Narita et al. Mol. Cell 2021) - this is now discussed in the manuscript. Future work may seek to look at e.g. transient chromatin-TF dynamics and in that case a more explicit representation of the full model may be necessary.

*The assumption of $Q = \Xi$*: The assumption that $Q = \Xi$ is not essential for our analysis, as demonstrated in the Methods section, where most of our derivations consider the correlated case. I have now also performed an analysis on the effect of asymmetry through the noising of $\Xi$ (see also the response to Referee 2), demonstrating the robustness of the conclusions to such asymmetry. The $Q = \Xi$ assumption is useful as it allows us to directly simulate and analyse model dynamics without explicit knowledge of enhancer parameters, and it strengthens connections to artificial neural network models. However, in some cases, such as our section on cell type evolution, a more general model is necessary. I have clarified these distinctions in the revised manuscript.

*The assumption of large $\beta$* in the manuscript means that we are considering the case of $\beta \to \infty$.

Practically, we take a finite $\beta$ that is large enough for the rows to all be fixed points, which depends on the dimensionality of $\beta$ and the correlations between the different cell types. This is clarified in the revisions. We also added a new methods section that provides an estimate for the gain in $\beta$ required to stabilise a new cell type from a multipotent progenitor (see reply to Referee 2).

*The assumption that the rows of $\Xi$ have identical magnitude* (e.g. unit) is also not essential for our conclusions, and we expand on this in the revised manuscript. This assumption is important in a specific case: if two rows $\Xi_i$ are similar ($\Xi_i$, $\Xi_j$ have small angular distance) and their difference in magnitudes is substantial, then at high $\beta$ only the higher-magnitude row will be stabilised. The lower magnitude row will therefore not be an attractor of the dynamics at any $\beta$. It is therefore useful to assume that rows corresponding to different stabilised cell types have similar magnitudes, which we set to unity without loss of generality. This assumption is appropriate due to the following reasons:

1. The rows $\Xi_i$ correspond to biochemical rates set by enhancer features such as the density and organisation of binding motifs, and potentially other features such as overall accessibility of the enhancer to transcription factor binding. These may evolve to have comparable overall magnitudes between competing cell types.
2. Alternatively, some parameters, such as overall chromatin accessibility at an enhancer, may be under the control of signalling factors. For example, we may consider the possibility that overall binding, corresponding to a gain parameter that scales the magnitude of $\Xi_i$, is controlled by signalling. These signalling factors may be tuned by negative feedback at the cell population level and thus at steady-state conditions where there is balanced production of different cell types, the effective magnitudes will be comparable.

*Taking **w**=0 in the direct reprogramming section*: As discussed in the manuscript, the main role of **w** is to set the baseline energy levels of different states, and to function downstream of signalling. In the absence of any prior knowledge about **w** (which may, for example, be affected by media conditions), setting **w**=0 would correspond to all cell types having comparable accessibility. We show that this assumption is not important for our conclusions in the reprogramming section, which rather depend on the expression profiles of the terminal cell identities, as we demonstrate in the revised analysis of direct reprogramming in the Methods section which is performed for a general **w**.

The revised model derivation section, now placed in Methods, discusses modelling assumptions including transcriptional machinery multiplicity, Boltzmann weights assumption, and quasi-steady-state assumption:

\subsection*{Derivation of general EnhancerNet model}
We modelled transcriptional activation by considering a process whereby transcription is initiated at enhancer $i$ at rate $p_i$, which results in the transcription of gene $j$ by a coupling rate $q_{i,j}$. As in established thermodynamic models for gene regulation, we assumed that there is

© 2024. Published by The Company of Biologists under the terms of the Creative Commons Attribution License (https://creativecommons.org/licenses/by/4.0/).

a rate limiting step for transcription which involves the recruitment to a specific site in the genome of a transcription initiation machinery \cite{ackers1982quantitative,bintu2005transcriptional}, which may be of high multiplicity (the effect of multiplicity in the model would be to scale overall transcription, which is not important for our conclusions). We assume that enhancers compete for the binding of this machinery. We denote by $\epsilon_i$ the energy for transcriptional initiation at enhancer $i$, and assume that the transcriptional machinery is always bound to one of the enhancers. We assumed that on the timescale of interest, which is related to changes in cell identity (days-weeks), the rate of transcription initiation is captured by equilibrium statistical mechanics. Namely, the rate of transcription initiation at enhancer $i$ is proportional to the Boltzmann distribution:
\begin{equation}
p_i = \frac{e^{-\hat{\beta} \epsilon_i}}{\sum_l e^{-\hat{\beta} \epsilon_l}} = \textrm{softmax} (-\hat{\beta} \epsilon_i)
\label{dynBoltzmann}
\end{equation}
where $\hat{\beta}$ is inverse temperature.
The initiation of transcription at enhancer $i$ can then result in the transcription of several associated genes, and each gene can be transcribed following an interaction with one of several enhancers. The dynamics of the expression of gene $j$, denoted $x_j$, is given by the sum:
\begin{equation}
\tau \dot{x_j} = \sum_i q_{i,j} p_i - x_j
\end{equation}
where $\tau$ is the timescale of gene expression changes.

Feedback in the model occurs when TFs modulate the activation energies of the enhancers. In classical models for gene regulation in prokaryotes, the binding of activator or repressor molecules directly modulates the rate of transcription initiation, corresponding to the variables $\epsilon_i$ in our model \cite{bintu2005transcriptional}. For enhancer-mediated transcription, on the other hand, the dominant mode of enhancer activation appears to be \emph{indirect}, through the modulation of enhancer chromatin \cite{eck2020quantitative}. Binding of TFs to enhancers leads to loosening of nucleosomes and to the biochemical modification of histone proteins \cite{calo2013modification,park2021interplay,hansen2022test}, resulting in dynamic changes in enhancer chromatin that are closely linked to enhancer activity \cite{heintzman2009histone, creyghton2010histone}. A dominant mode of enhancer activation appears to be the TF-mediated recruitment of histone acetyltransferases, which modify enhancer chromatin, resulting in the recruitment of transcription initiation machinery \cite{narita2021enhancers}.

From a biophysical perspective, this can be captured by a model in which $\epsilon_i$ is set by a latent variable $m_i$, which accumulates proportionally to the binding of TFs:

\begin{equation}
\epsilon_i = -\hat{w_i} - m_i
\end{equation}
\begin{equation}
\dot{m_i} = \kappa_1 \sum_j {\xi_{i,j}x_j} - \kappa_{-1} m_i \\
\label{eq:enmod}
\end{equation}
where $\xi_{i,j}$ is the effective binding rate TF $j$ to enhancer $i$, and where $\kappa_1,\kappa_{-1}$ are the associated "on" and "off" rates for $m_i$, which may be related to the global activity of enzymes such as histone acetyltransferases and histone deacetylases. Experimentally, it was observed that the timescale of these dynamics is on the order of minutes \cite{weinert2018time,narita2021enhancers}, and thus much faster than the typical timescale of changes in cell identity. The weight parameter $\hat{w_i}$ determines the energy in the absence of $m_i$, or effects on $m_i$ that are outside the cell identity feedback network.

The mechanism described in Eq.~\ref{eq:enmod} is generic and may correspond to several underlying biological processes and is, in essence, similar to modulation of receptor activation energy by methylation in bacterial chemotaxis \cite{tu2013quantitative}. Taking a quasi-steady-state of $m_i$ and denoting by $\kappa=\frac{\kappa_1}{\kappa_{-1}}$, we derive

© 2024. Published by The Company of Biologists under the terms of the Creative Commons Attribution License (https://creativecommons.org/licenses/by/4.0/).

Eq.~\ref{eq:matdyn}.

Note that it is also possible to consider a more general model where TF binding to enhancers is modulated at the level of the entire enhancer, for example by the binding of "pioneer" factors that increase accessibility for other TFs. In such a case, Eq.~\ref{eq:enmod} may be altered so that:
\begin{equation}
\dot{m_i} = \kappa_1 \zeta_i \sum_j {\xi_{i,j}x_j} - \kappa_{-1} m_i \\
\label{eq:enmodB}
\end{equation}
where $\zeta_i$ corresponds to enhancer-level gain, which effectively multiplies $\Xi_i$.

The revised *Methods* section where we discuss and quantify the significance of comparable row magnitudes:
Consider the row vectors of $\Xi$ given by $\Xi_i = \left(\xi_{i,1},\dots,\xi_{i,N}\right)$. The dynamics of Eq.~\ref{eq:enhancernetsym} at $\textbf{x}=\Xi_k$
are given by:
\begin{equation}
\begin{split}
\tau \dot{x_j} &= \sum_i {\xi_{i,j} \frac{e^{w_i +\beta \sum_r {\xi_{i,r}\xi_{k,r}}}}{\sum_l e^{w_l +\beta \sum_r {\xi_{l,r}\xi_{k,r}}}}} - \xi_{k,j} \\
&= \sum_i {\xi_{i,j} \frac{e^{w_i +\beta \langle\ \Xi_i,\Xi_k\rangle\ }}{\sum_l e^{w_l +
\beta\langle\ \Xi_l,\Xi_k\rangle\ }}} - \xi_{k,j}\\
&= \sum_i {\xi_{i,j} \frac{e^{w_i +\beta || \Xi_i || || \Xi_k || \cos{\theta_{i,k}} }}{\sum_l e^{w_l + \beta || \Xi_l || || \Xi_k || \cos{\theta_{l,k}} }}} - \xi_{k,j}
\end{split}
\end{equation}
where $\theta_{i,j}$ determines the angle between $\Xi_i, \Xi_j$ and the magnitude is defined by the Euclidean norm. We assume that the rows of $\Xi$ are all of magnitude unity, which is equivalent to assuming that they have comparable overall binding affinities.
In this case:
\begin{equation}
\begin{split}
\tau \dot{x_j} &= \sum_i {\xi_{i,j} \frac{e^{w_i +\beta \cos{\theta_{i,k}} }}{\sum_l e^{w_l + \beta \cos{\theta_{l,k}} }}} - \xi_{k,j}
\\ &= \xi_{k,j} - \xi_{k,j} = 0
\end{split}
\end{equation}
where the last equality holds for sufficiently large $\beta$ and assuming the rows of $\Xi$ are distinct, since in that case $\cos{\theta_{i,k}}=1$ if and only if $i=k$. Thus, for a large enough $\beta$, the rows of $\Xi$ are fixed points of the dynamics.
In practice, it is only important that cell types with high cosine similarity have similar overall binding affinities. Consider, for example, the case where, for pattern $k'$, we have that:
\begin{equation}
|| \Xi_{k'} || > \frac{|| \Xi_k || }{\cos{\theta_{k',k}}}
\end{equation}
In such a case, the pattern $k$ will be unstable even at very large $\beta$, and thus such a pattern will not be accessible. For this reason, we expect that similar cell types will have comparable overall binding affinities. In cells, we expect that this could be achieved either through evolution of enhancer composition, or by population-level negative feedback, such as the coupling of the enhancer-level gain parameter ($\zeta_i$) to the production of cell type $i$.

What about the case where the matrix $\textbf{Q}$ is distinct from $\Xi$? Consider then dynamics at
$\textbf{x}=\textbf{Q}_k$:
\begin{equation}
\begin{split}
\tau \dot{x_j} = \sum_i {q_{i,j} \frac{e^{w_i +\beta || \Xi_i || || \textbf{Q}_k ||
\cos{\theta_{i,k}} }}{\sum_l e^{w_l + \beta || \Xi_l || || \textbf{Q}_k || \cos{\theta_{l,k}} }}} -

© 2024. Published by The Company of Biologists under the terms of the Creative Commons Attribution License (https://creativecommons.org/licenses/by/4.0/).

q_{k,j}
\end{split}
\end{equation}
where now $\theta_{i,j}$ determines the angle between $\Xi_i, \textbf{Q}_j$. Now, in order for the pattern $\textbf{Q}_k$ to be a fixed point at high $\beta$, we require that:
\begin{equation}
|| \Xi_k || || \textbf{Q}_k || \cos{\theta_{k,k}} = \max_i {|| \Xi_i || || \textbf{Q}_k ||
\cos{\theta_{i,k}} }
\end{equation}
Under the assumption that the rows of $\Xi$ are comparable, we have that $\textbf{Q}_k$ is a fixed point of the dynamics at sufficiently large $\beta$ when there is high cosine similarity between $\Xi_k, \textbf{Q}_k$ compared with the other rows of $\Xi$.

The new Methods section where I estimate the gain in $\beta$ required to co-stabilize similar cell types:

\subsection*{Temperature transitions for cell type evolution}

In this section, we will estimate the temperature $\beta$ required to specify a population of related cell types. These cell types may share activity patterns across many of their TFs and differ in some smaller portion. As an example that will be discussed later, neurons may have similar TF activity patterns across many TFs, yet small differences in TF activity can fine-tune the identity of specific neurons.

We will specifically consider $K$ patterns associated with closely related cell types and $K'$ patterns associated with other cell types. There are $N$ bits that differ between the related cell types, of which a fraction $\eta$ are active (drawn at random), $N''$ bits that are inactive, and $N'$ bits that are active in all other cell types. Since patterns have magnitude unity, we set the activity level of active bits to $a=\frac{1}{\sqrt{\eta N+N'}}$. We assume that unrelated patterns have $\eta N + N'$ active TFs drawn at random.

The average inner product of the related cell types is given by:
\begin{equation}
A=\frac{\eta^2 N + N'}{\eta N + N'}
\end{equation}

While the average inner product between random cell types is given by:
\begin{equation}
B = \frac{\eta N + N'}{N + N'' + N'}
\end{equation}

Let $\Xi_1,\dots,\Xi_{K}$ denote each of the closely related patterns, and $\bar{\Xi}$ their average. The energy of each pattern is given by:

\begin{equation}
V_1 = V(\Xi_1) \approx -\beta^{-1}\log{\left(e^{\beta} + (K-1){e^{\beta A} + K' e^{\beta B} }\right)} + \frac{1}{2}
\end{equation}
We will assume that the patterns are sufficiently well separated ($A\gg B$) and $\beta$ sufficiently large such that:
\begin{equation}
V_1 \approx -\beta^{-1}\log{\left(e^{\beta} + (K-1){e^{\beta A} }\right)} + \frac{1}{2}
\end{equation}
while the energy of the averaged pattern is:
\begin{equation}
V_2=V(\bar{\Xi})\approx -\beta^{-1}\log{K} - \frac{A}{2}
\end{equation}

© 2024. Published by The Company of Biologists under the terms of the Creative Commons Attribution License (https://creativecommons.org/licenses/by/4.0/).

In general, as $\beta$ increases, we expect to see first a transition from the global average to the local average of the two patterns, which occurs when $V_1$ drops below $V_2$. This transition occurs at a critical $\beta$ that satisfies (assuming $K\gg 1)$:
\begin{equation}
-\frac{1}{\beta} \log \left(e^{\beta (A-1)}+\frac{1}{K}\right)=\frac{1}{2}-\frac{A}{2}
\end{equation}

When $\beta$ is small then $e^{\beta (A-1)}\gg\frac{1}{K}$ and the l.h.s is equal to $1-A$ and thus larger than the r.h.s. When $e^{\beta (A-1)}\ll\frac{1}{K}$ the l.h.s is negative and thus smaller than the r.h.s. Thus, the critical $\beta$ occurs around:
\begin{equation}
\beta_{\text{crit}_1} \approx \frac{1}{1-A}\log{K} = \frac{1}{1-\eta } \left(\frac{N'}{\eta N}+1 \right)\log{K}
\label{eq:critA}
\end{equation}

We will now consider the case where a new cell type $\Xi_1'$ evolves from $\Xi_1$. We will assume, without loss of generally, that the evolved pattern $\Xi_1'$ differs from the existing pattern $\Xi_1$ by two bits, namely $\xi_{1,1}=a,\xi_{1,1}'=0,\xi_{1,2}=0,\xi_{1,2}'=a$.
The energy of the new patterns is given by:

\begin{equation}
V_3=V(\Xi_1')= V(\Xi_1) \approx -\beta^{-1}\log{\left(e^{\beta}+e^{\beta C}+ (K-1){e^{\beta A} }\right)} + \frac{1}{2}
\end{equation}
with $C$ corresponding to the inner product of the new cell types:
\begin{equation}
C = 1-\frac{1}{\eta N + N'}
\end{equation}
Whereas the energy of the averaged pattern is:
\begin{equation}
V_4 = V\left(\frac{1}{2}\left(\Xi_1'+\Xi_1\right)\right) \approx -\beta^{-1}\log{\left(2 e^{\beta C}+(K-1){e^{\beta A} }\right)} + \frac{C}{2}
\end{equation}
For the second transition, we are interested in $\beta>\beta_{\text{crit}_1}$ where the contributions of the $(K-1){e^{\beta B}}$ terms to the energy levels are negligible. In this case, we can use Eq.~\ref{eq:bipotent}:
\begin{equation}
\beta_{\textbf{crit}_2} = (\eta N + N') \log{4}
\label{eq:bipotentB}
\end{equation}
Using Eqs.~\ref{eq:bipotentB},\ref{eq:critA} we can derive a ratio $\chi$ that captures the relative increase in $\beta$ required to evolve and stabilize a new cell type from an existing population of cells:
\begin{equation}
\chi= \frac{\beta_{\textbf{crit}_2}}{\beta_{\textbf{crit}_1}} = \frac{(1-\eta ) \eta N \log{4}}{\log{K}}
\end{equation}
the gain $\chi$ thus only depends on the TFs that are variable between the cells, and, amongst these TFs, scales with the variance of the active TFs.
For the case of neuronal identity specification in \emph{C. elegans}, the expression code is sparse, with $\mu=0.1$ among variable TFs, with the other parameters being $K=118$ neuron classes and $N=68$ TFs exhibiting variable expression across neuronal classes. For this system, we thus have that:
\begin{equation}
\chi= \frac{\beta_{\textbf{crit}_2}}{\beta_{\textbf{crit}_1}} = \frac{(1-\eta ) \eta N \log{4}}{\log{K}} = 1.8
\end{equation}
implying a further 80\% increase in $\beta$ required from the initial destabilization of a multipotent progenitor to the complete specification of all cell types.

© 2024. Published by The Company of Biologists under the terms of the Creative Commons Attribution License (https://creativecommons.org/licenses/by/4.0/).

New supplementary figure testing asymmetry in TF-enhancer interactions:

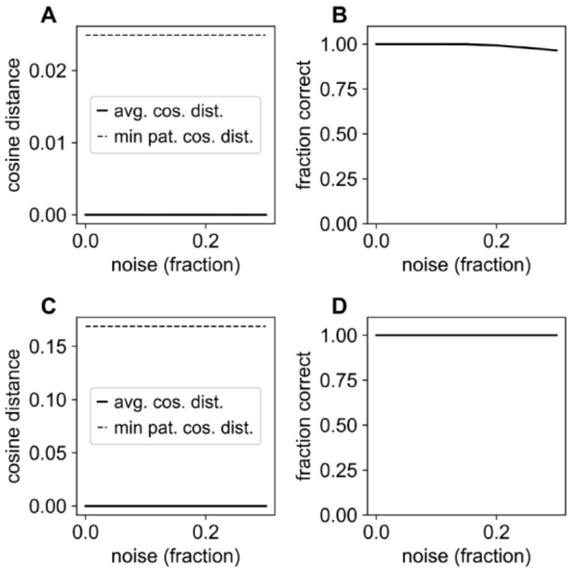


\subsection*{Figure S1. Robustness of fixed points to asymmetry in enhancer-TF interactions (Related to Figure 2).}

Throughout the manuscript, we assume symmetry in enhancer-TF interactions, captured mathematically by $\textbf{Q}=\Xi$. However, in practice, it is likely that the matrices are correlated but not perfectly identical, that is, there is some asymmetry in the dynamics. To test for the effect of this asymmetry, we added noise to the entries of $\Xi$ by multiplying each entry by a scalar drawn from a normal distribution with mean $1$ and standard deviation ranging from $0$ to $0.3$ (entries were capped at a minimum of $0$) while fixing the \textbf{Q} matrix. We used the $\Xi$ matrix based on the Tabula Muris data (A,B) or a matrix initialized with uniformly drawn entries of the same dimensions (C,D). As in Figure 2, the dynamics were initialized from each of the rows of $\textbf{Q}$. Even at very high noise magnitudes, the dynamics always settle at a cell type specified by the $\textbf{Q}$ matrix (A,C, solid line denoting average minimal cosine distance of final state from rows of $\textbf{Q}$, dashed line minimal cosine distance between rows of $\textbf{Q}$). The only effect of noising is to destroy specific attractors of very similar cell types, such as T/NK cells, by destroying their distinguishing features (B,D, line capturing average fraction of final states that are identical to the initial state).

Revised *Methods* section on direct reprogramming, which consider a general **w**:

\subsection*{Direct reprogramming through the over-expression of TFs}
Consider a transcription factor $x_j$ that is constitutively overexpressed to an extent $\delta_j$. This constitutive expression alters energy levels $\epsilon_i$ without feedback. We therefore consider only the effect of the additional TF production on the energy levels of the enhancers, without altering the coordinates of the stable fixed points. We denote by $V'$ the potential under the perturbed dynamics. Evaluating $V'$ at pattern $k$ yields, at high $\beta$:
\begin{equation}
\begin{split}
V'(\Xi_k)&=-\beta^{-1}\log{\sum_i {e^{w_i + \beta\left({|| \Xi_i || || \Xi_k || \cos{\theta_{i,k}}}
+ \xi_{i,j} \delta_j \right) }}} + \frac{1}{2} \Xi_k^T\Xi_k \\
&=-\beta^{-1}\log{\sum_i {e^{w_i + \beta\left({\cos{\theta_{i,k}}} + \xi_{i,j} \delta_j \right)
}}} + \frac{1}{2}
\end{split}
\end{equation}
where we assumed that $|| \Xi_i ||=1$ for all $i$. Assuming that uperturbed pattern $k$ is

© 2024. Published by The Company of Biologists under the terms of the Creative Commons Attribution License (https://creativecommons.org/licenses/by/4.0/).

stable at a given $\beta$, we have that the summand for $\delta_j=0$ takes its maximum at $i=k$, where:
\begin{equation}
\sum_{i} e^{w_i + \beta \cos{\theta_{ik}} }\approx e^{w_k+\beta}
\end{equatio
n} And thus:
\begin{equation}
V(\Xi_k) = -\frac{w_k}{\beta} - \frac{1}{2}
\end{equation}

Under the assumption that the perturbation magnitude is relatively small (and, generally, for patterns that are stable after the perturbation), we have that:
\begin{equation}
\sum_{i} e^{w_i+\beta \left(\cos{\theta_{ik} + \xi_{i,j} \delta_j} \right)}\approx
e^{w_k+\beta(1+\xi_{k,j}\delta_j)}
\end{equatio
n} and thus:
\begin{flalign}
V'(\Xi_k) &= -\frac{w_k}{\beta} - \frac{1}{2}-\xi_{k,j}\delta_j = V'(\Xi_k) -\xi_{k,j}\delta_j
\label{eq:reprogramming}
\end{flalign}
Note that Eq.~\ref{eq:reprogramming} readily generalizes to a perturbation in several TFs $j\in\mathcal{J}$:
\begin{flalign}
V'(\Xi_k) &= V(\Xi_k) - \sum_{j\in\mathcal{J}}{\xi_{k,j}\delta_j}
\label{eq:reprogrammingB}
\end{flalign}
If the goal is to steer the dynamics toward a pattern $k$ then the TFs $\mathcal{J}$ and their overexpression need to be chosen such that $\sum_{j\in\mathcal{J}}{\delta_j\xi_{i,j}}$ is sufficiently larger for $i=k$ than for all other patterns $i\neq k$. That is, the ideal expression for a TF $j$ is such that $\xi_{i,j}$ is large for $i=k$ (amplifying $\delta_j$) and zero for $i\neq k$.

For the hematopoiesis simulations, the author needs to specify more clearly how this was actually implemented. Does the homogeneous initial population mean that the initial conditions are the same for all cells or that the initial conditions homogeneously explored the parameter space? In addition, noise was added to the dynamics: how important is this noise for the generation of the correct lineages? What is the size of the noise and is that biologically realistic? How sensitive are the results (particularly the proportions) to these fluctuations? Also I do not understand the biological rationale for the negative feedback routine used to tune the weight vectors.

In the revised manuscript, I have improved the presentation of the hematopoiesis simulations to address the issues raised by the referee. I now explain that the initial conditions are not important because the transition to low $\beta$ in the annealing process entails a uniform transition to a single global steady-state corresponding to a multipotent progenitor cell type. The role of noise in the simulations is to allow the production of different cell fates from similar initial conditions during the annealing process, enabling multiple fates to arise from multipotent progenitors (otherwise on a single cell fate would arise). The magnitude of the noise is not important for our key results, namely the structure of the differentiation landscape including the existence and identity of progenitor states, as these are set by the deterministic part of the dynamics. In the revisions, we demonstrate this by showing that our results remain effectively unchanged when varying noise amplitude by an order of magnitude (Figure S3).

I have also improved the presentation of the feedback procedure and the rationale behind the feedback tuning mechanism. The main idea is as follows: In systems such as hematopoiesis, where a single stem/progenitor population can differentiate into multiple terminal cell types, the rate of differentiation is set by negative feedback from various intercellular factors. For example, the concentration of EPO biases erythroid cell fates, which in turn decreases the rate of EPO secretion. In our model, the interpretation of such signalling is that it modulates $\mathbf{w}$, which then controls the relative fraction of cell types produced in the annealing procedure. By implementing the negative feedback procedure, I can thus simulate physiological feedback regulation of cell

© 2024. Published by The Company of Biologists under the terms of the Creative Commons Attribution License (https://creativecommons.org/licenses/by/4.0/). 

fate control and produce balanced differentiation trajectories.

The revised Results section presenting the hematopoiesis analysis:

The model makes the specific prediction that differentiation hierarchies, including the identity of observed progenitor states, can be estimated by only knowing the identities of the terminal cell states. The model specifically predicts that they will appear only between transcriptional profiles with high internal cosine similarity. To test this, we considered differentiation in hematopoiesis, a system in which differentiation trajectories are complex and have been extensively studied. Using data on hematopoietic lineages in mice (extracted from Haemopedia \cite{choi2019haemopedia}), we considered the expression profile of all TFs that showed variability between terminal lineages and used the model to estimate the differentiation hierarchy that generated these lineages.

We first tested the hypothesis that progenitors emerge between correlated expression profiles by performing hierarchical clustering according to pairwise cosine similarity between the expression profiles of terminal cell types (Figure 3C). Hierarchical clustering provides a heuristic approach to estimate the predicted identity of progenitor states from the terminal expression profiles. It produces a dendrogram (tree) that shows how expression profiles are progressively grouped into clusters based on their similarity. This resulted in a tree structure that recreates the classic hematopoietic hierarchy (Figure 3C). Branching points at the dendrogram capture the Common Lymphoid Progenitor (CLP), Common Myeloid Progenitor (CMP), Megakaryocyte and Erythroid Progenitor (MEP), Granulocyte and Macrophage Progenitor (GMP), T and NK cell progenitors (TNK), and B cell-biased lymphoid progenitor (BLP). Thus, a simple unsupervised clustering method with access to only the terminal cell fate information can reproduce a faithful estimate of the known complex differentiation landscape in hematopoiesis.

Motivated by the effectiveness of hierarchical clustering in predicting progenitor states, we developed a computational approach to model differentiation into multiple cell types. This approach is based on annealing in Eq.~\ref{eq:enhancernetsym}. We simulated annealing trajectories beginning with a homogeneous initial population representing hematopoietic stem cells. To account for natural variability in differentiation paths, we introduced noise to Equation \ref{eq:enhancernetsym} (our findings remain robust across various noise magnitudes, Figure S3). In physiological settings, the production rate of each cell type is regulated by negative feedback, where the terminal cell population size inhibits its own production. This is exemplified by the negative feedback regulation of red blood count through EPO signalling \cite{grover2014erythropoietin}. To incorporate this phenomenon, we implemented a negative feedback routine to tune $\textbf{w}$, allowing each cell type to inhibit its own production (Methods). This resulted in dynamics that generated all cell fates in a balanced manner. We then visualized the differentiation trajectories on a UMAP plot (Figure 3D), revealing that differentiation dynamics proceed through a series of multipotent progenitors corresponding to those observed in hematopoiesis. Thus, our model quantitatively recapitulates differentiation in hematopoiesis without requiring parameter fitting.

While the heuristic approach of hierarchical clustering produces a tree-like structure that recapitulates the main progenitor states in hematopoiesis, differentiation trajectories in the model need not be tree-like, as can be seen in the simulations of Figure 3D. As $\beta$ increases, multiple steady states can appear and disappear, and these new steady states may be averages of overlapping terminal states. The key prediction of the model is that these states will correspond to averages of terminal states with high internal similarity. For example, it is experimentally established that dendritic cells can emerge from both the myeloid lineage through macrophage-dendritic progenitors and from the lymphoid lineage through B cell-biased lymphoid progenitors \cite{anderson2021genetic}. This observation aligns with our model and simulations (Figure 3D, see also Figure S4 for simple examples of tripotent progenitors and deviations from tree structure). The gene expression profile of dendritic cells exhibits high cosine similarity to both B cells and macrophages (and, more generally, myeloid cells), while B cells are less similar to myeloid cells. Similarly, neutrophils may arise from distinct macrophage-neutrophil and basophil-eosinophil-neutrophil progenitors, and macrophages from distinct macrophage-neutrophil and macrophage-dendritic cell progenitors \cite{weinreb2020lineage,yampolskaya2023sctop}. These patterns are also recapitulated in our simulations and are related to the similarity structure of

© 2024. Published by The Company of Biologists under the terms of the Creative Commons Attribution License (https://creativecommons.org/licenses/by/4.0/). 

the cell types. Higher-level progenitor structures can be captured in the same manner by the model. For example, lymphoid and granulocyte cells have moderate cosine similarity, as do granulocyte and erythro-megakaryocytes. However, lymphoid and erythro-megakaryocytes are poorly correlated. Consequently, each of the first two pairs can share accessible progenitors, while the latter is less likely to do so. Indeed, the first two progenitors exist (Lympho-Myeloid Primed Progenitors and Common Myeloid Progenitors), while the latter, to the best of our knowledge, does not. The model can thus make predictions regarding highly complex transition dynamics by only using information on the terminal cell identities.

The revised Methods section on the approach:

\subsubsection*{Simulation of balanced differentiation}
Hematopoiesis was simulated by using annealing from $\beta=0$ to $\beta=50$ over 50 time units. The initial state corresponded to the averaged value of all terminal states. Simulations were performed by adding additive white noise:
\begin{equation}
dx = \left(\Xi^T \textrm{softmax}\left(\textbf{w}+\beta\Xi \textbf{x} \right) - \textbf{x} \right) dt + \sigma dW
\end{equation}

Where we set $\sigma=0.01$. To simulate balanced differentiation in hematopoiesis we used the following feedback procedure that aims to imitate \emph{in vivo} feedback on differentiation, where there is negative feedback on the production of each cell type by signalling. $\textbf{w}$ was initialized as a zero vector. Then, running from $k=0$ to $k=k_{\text{max}}$, we performed differentiation by annealing. If the outcome terminal cell corresponded to row $i$ of $\Xi$, we adjusted $w_i=w_i-0.5(1-k/k_{\text{max}})$, and then mean-adjusted \textbf{w} to zero. This resulted in a signalling profile $\text{w}$ which produced all cell fates from the initial progenitor population.

The new Figure S3, where we tested sensitivity to noise magnitude:

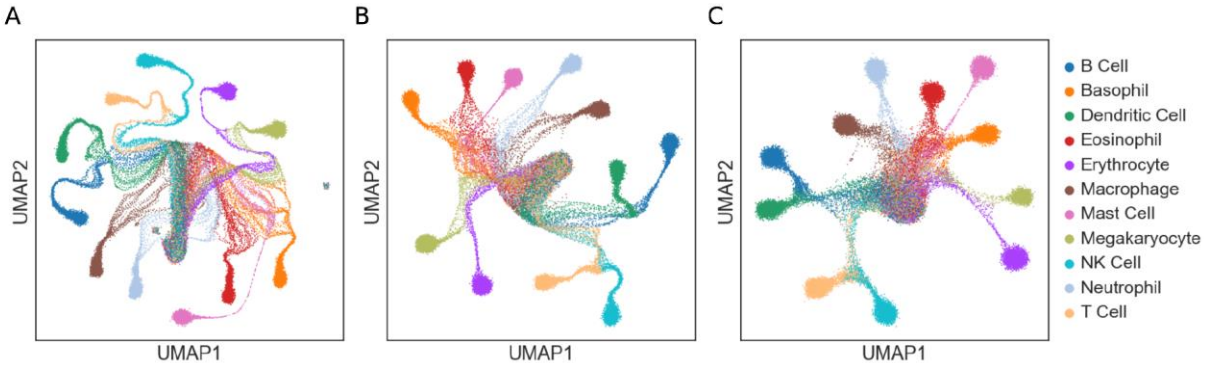


\subsection*{Figure S3. Blood differentiation with different noise magnitudes (Related to Figure 3).}

Differentiation in hematopoiesis, as in Figure 3D, was simulated by taking (A) $\sigma=0.003$, (B) $\sigma=0.01$, (C) $ \sigma=0.03$, and then plotted as in Figure 3D.

It would also be helpful if the author could comment on other mechanisms for establishing stable cell type expression states. In the EnhancerNet scheme this results from fixed points of a set of TF/enhancer differential equations. But there are other possibilities where, for example, the chromatin itself locally specifies memory of the transcriptional state (through independent feedback involving Polycomb or heterochromatin or DNA methylation). How would that interface with the author’s scheme?

I agree with the referee that mechanisms involving local memory, such as bistability of local chromatin state, may play an important role in specifying and reinforcing transcriptional states. These mechanisms are now discussed in the revised manuscript. While a full analysis of such mechanisms and their interface with our model is beyond the scope of this study, the revised

© 2024. Published by The Company of Biologists under the terms of the Creative Commons Attribution License (https://creativecommons.org/licenses/by/4.0/). 

manuscript presents an exploration of one important possibility: that local memory mechanisms at enhancers can contribute to the stabilisation of cell types and provide barriers to reprogramming. The efficacy of this mechanism is uniquely due to the association between enhancer activity and cell type specification.

The revised Discussion paragraph:

Other mechanisms in addition to histone acetylation are known to play a role the regulation of enhancer activity. These include the activity of chromatin remodellers such as the SWI/SNF, Mi-2/NuRD, SET1/MLL, and Polycomb complexes, and involve various mechanisms including the depletion and modification of histone proteins \cite{gao2009opposing,whyte2012enhancer,iurlaro2021mammalian,wolf2023cooperation,chan2018po lyc omb}. In addition to these, the methylation of DNA itself can play a role in regulating enhancer function \cite{angeloni2019enhancer}. Some epigenetic mechanisms may show bistability through local positive feedback regulation, resulting in digital activation patterns \cite{dodd2007theoretical,angel2011polycomb,haerter2014collaboration,berry2017slow,sneppen201 9theo retical,movilla2024two}. This property may confer robustness to cell types and protect against spontaneous reprogramming. Specifically, we considered the case where enhancer activity acts as a bifurcation parameter for a bistable switch that inhibits enhancer activity (Methods). In such a case, a drop in enhancer activity below a critical threshold results in positive feedback that further decreases the basin of attraction for the corresponding cell type, resulting in a barrier for reprogramming.

The new Methods section:

\subsection*{Local positive feedback as a barrier for reprogramming}

Consider a modification to the EnhancerNet model where the energy for transcriptional activation is dependent on both the original modification $m_i$ and a new modification $u_i$:
\begin{equation}
\epsilon_i = -\hat{w}_i - m_i + \lambda u_i
\end{equation}
Here, $\lambda$ is a scalar and $u_i$ is autocatalytic and inhibited by enhancer activation. We propose the following dynamics for $u_i$:
\begin{equation}
\dot{u_i} = f(p_i) + \rho \frac{u_i^n}{1+u_i^n} - u_i
\label{eq:bistable}
\end{equation}
where $n\gg 1$ and $f(p_i)$ is a decreasing function of enhancer activity $p_i$. Equation \ref{eq:bistable} can exhibit either bistability or hysteresis, depending on the value of $\rho$.
We assume that initially $u_i=0$ and $p_i$ is sufficiently large (as in the multipotent state), maintaining stability at $u_i=0$. As cells differentiate, the activity of specific enhancers decreases while others increase. When the activity of an enhancer drops below a critical threshold $p_{\text{crit}}$, Equation \ref{eq:bistable} undergoes a bifurcation, causing $u_i$ to increase to a much larger value ($u_i\gg 1$). This increase may persist due to a transition to a new fixed point when the dynamics are bistable.
Consequently, the energy required to initiate transcription from the enhancer increases, making reprogramming to the associated cell type (whether induced by signalling, overexpression of specific transcription factors, or noise) less likely. This mechanism effectively creates a barrier to reprogramming.

**Reviewer 2.**
In his manuscript “A model for enhancer selection recapitulates the dynamics of cell type acquisition”, Karin proposes that the essential dynamcis of cell fate networks can be mathematically described using “Modern Hopfield Networks”. He also proposes a mechanistic explanation for these dynamics based on enhancers and TF interactions. The work is theoretically sophisticated and quite compelling. I think the paper is very good and ideally suited for a journal like Development.

Thank you for this strong endorsement of the manuscript.

© 2024. Published by The Company of Biologists under the terms of the Creative Commons Attribution License (https://creativecommons.org/licenses/by/4.0/).

My main comments regard: connection to earlier literature, minor clarifications and extensions, and numerically testing how robust the construction is to introducing asymmetry in the TF-enhancer interactions.

The theoretical starting point for this work is the idea that cell fates can be viewed as attractors of a dynamical system. It is builds upon an earlier works, that the author is likely not aware of, that Hopfield-inspired attractor networks offer a compelling model of cell fate (see discussion of this in this review Teschendorff et al. Nature Reviews Genetics 22.7 (2021): 459-476.; Lang et al. PLoS computational biology 10.8 (2014): e1003734.; Pusuluri et al. Physical Biology 15.1 (2017): 016001.; Smart et. al. Cell Reports Physical Science 4.2 (2023). Yampolskaya, Maria"scTOP: physics-inspired order parameters for cellular identification and visualization." Development 150.21 (2023): dev201873.). The authors independently arrive at a similar model, but with three very interesting and important extensions. First, instead of using ordinary Hopfield networks they extend this construction to Modern Hopfield networks. Second, they give a mechanistic interpretation of these models in terms of enhancer-TF interactions. Third, they argue that the spurious attractors in these networks can be naturally interpreted as progenitor cell types. It would be nice if the authors could connect the current results with this earlier literature and discuss the implications of these differences for predictions.

We thank the referee for providing these very important references. I have now extensively revised the introduction section in the manuscript to properly reference and present these pioneering works, which indeed provide compelling motivation to pursue Hopfield-like models for studying cell fate regulation. I also discuss how the present work connects with previous work, how it extends it, and the differences for predictions:

The importance of TFs is reflected in their central role in models of cell identity dynamics. Classical models focused on how small transcriptional network motifs, involving just a few TFs, can generate cell fate bifurcations, such as those specific to blood lineages \cite{ferrell2002self,huang2007bifurcation,alon2007network,graham2010modeling,saez2022statistically}. While these models can be useful for understanding gene expression dynamics at specific decision points, they cannot predict larger-scale dynamics of the cell identity network, such as the complex, hierarchical differentiation patterns or reprogramming between distinct cell types.

In contrast to these "small network" models, pioneering work has demonstrated that aspects of cell identity gene network behaviour can be captured by high-dimensional attractor models based on Classical Hopfield networks \cite{lang2014epigenetic,fard2016not,pusuluri2017cellular,guo2017hopland,teschendorff2021statistical,y ampolskaya2023sctop,smart2023emergent}. Classical Hopfield networks are well-established models for dynamical systems that encode combinatorial attractor states (patterns) through the interactions of their components \cite{amari1972learning,little1974existence,hopfield1982neural}. In the context of transcriptional networks, the state of the dynamics is represented by the activity of cell identity TFs, with the attractor states corresponding to distinct cell types. In Hopfield networks, the entire dynamics of the system can be specified solely by defining the attractor states, which are the observed cell types. This allows for predictions about the gene regulatory network dynamics based on mere knowledge of the cell types encoded by the network. Lang et al. exploit this property to demonstrate that it is possible to capture and predict the effects of TF overexpression on cellular reprogramming \cite{lang2014epigenetic}. The Hopfield formulation also predicts that mixtures of attractor states may themselves be attractor states (known as "spurious attractors"). Lang et al. propose that this phenomenon explains the observed expression profiles of partially reprogrammed cells \cite{lang2014epigenetic}.

While this work provides a compelling motivation for applying attractor networks to study cell identity dynamics, it has several important limitations that restrict its applicability. First, while Hopfield dynamics provide an attractive computational model, the relation between this model and underlying molecular mechanisms for cell type specification is unclear. Another major drawback is the limited storage capacity of classical Hopfield networks, which can only encode a relatively small number of cell types given the available transcription factors. Crucially, these networks cannot stabilize correlated cell types, which are inherently prevalent in biological systems. The model also typically specifies binary expression patterns and does not capture

© 2024. Published by The Company of Biologists under the terms of the Creative Commons Attribution License (https://creativecommons.org/licenses/by/4.0/). 

continuous changes in expression levels, such as low-level multilineage priming in progenitor states. Finally, such progenitor states must be explicitly specified in the model as attractor cell types, rather than emerging naturally from the dynamics.

Here, we develop a theoretical framework for modelling the dynamics of the cell identity network by considering the feedback regulation of TFs through enhancers, which we denote as \emph{EnhancerNet}. Enhancers are regulatory elements crucial for cell identity specification. TFs bind to enhancers to activate cell-type specific expression patterns. Each enhancer can be bound by multiple TFs and, in turn, initiate gene expression in one or more distal target genes \cite{pennacchio2013enhancers,uyehara20233d}. The binding of TFs to an enhancer recruits cofactors that can alter its activity by modulating epigenetic properties, such as the biochemical characteristics of its associated chromatin. This, in turn, affects the transcription initiation rate associated with the enhancer \cite{calo2013modification,park2021interplay,hansen2022test,heintzman2009histone, creyghton2010histone}.

A well-documented experimental observation reveals a symmetry between TF binding and enhancer regulation. Specifically, TFs that determine cell identity bind to enhancers that regulate both their own expression and that of other identity-determining TFs coexpressed in specific cell types. This interaction forms dense autoregulatory networks \cite{hnisz2013super,whyte2013master,adam2015pioneer,saint2016models,feng2023landscape}. These dense autoregulatory networks likely play a crucial role in cell type specification, as their associated enhancers exhibit activation patterns that are highly cell type-specific \cite{hnisz2013super,heinz2015selection}. This cell type specificity of enhancer activity contrasts with the broader activity of TFs, which may be shared across multiple cell types \cite{hnisz2013super}.

We show that the symmetry between TF binding and enhancer regulation imposes an exacting constraint on the dynamics of the cell identity network model, resulting in a highly simplified dynamical model which is predictive and captures broad experimental observations on cell type specification, enhancer selection, differentiation through progenitors, and reprogramming, without the need of fitting unobserved parameters. We demonstrate a mathematical analogy between our mechanistic model and Modern Hopfield Networks \cite{krotov2016dense,ramsauer2020hopfield} which overcome the limitations of previous approaches. This analogy provides a mechanistic link between models for associative memories and cell identity networks, explains the role of distal regulatory elements with dynamic chromatin in multicellular evolution, and provides specific and testble predictions.

I found the reprogramming/differentiation experiments very interesting. I would like to know something about the trajectories that are found and how the energy (Lyapunov function) differs between reprogramming and differentiation. It was found in the earlier works above that the energy for reprogramming passed through high energy states whereas differentiation does not. Is this also the case here?

The reprogramming and differentiation trajectories are indeed consistent with this analysis. Our reprogramming trajectories are well-described by one-dimensional dynamics (see the new Figure S2), as discovered by Pusuluri et al. Due to the nature of our model, which incorporates continuous TF expression, we could actually observe a deterministic shift between cell types in reprogramming for a sufficiently large perturbation. For a smaller perturbation, and incorporating noise, reprogramming corresponds to stochastic barrier crossing as described by Pusuluri et al. In both cases, the transition would occur over what would otherwise be a high energy barrier.

Differentiation, on the other hand, occurs in our model through an annealing procedure that modulates the energy landscape. This approach differs from previous work, where progenitor states were encoded as distinct cell types. Our method offers an alternative interpretation of progenitor states as "transition states" that may be stabilized at intermediate β and exhibit multilineage priming. Nevertheless, our model aligns with Lang et al.'s conclusions in that energy levels decrease during the differentiation process.

The new Figure S2:

© 2024. Published by The Company of Biologists under the terms of the Creative Commons Attribution License (https://creativecommons.org/licenses/by/4.0/).

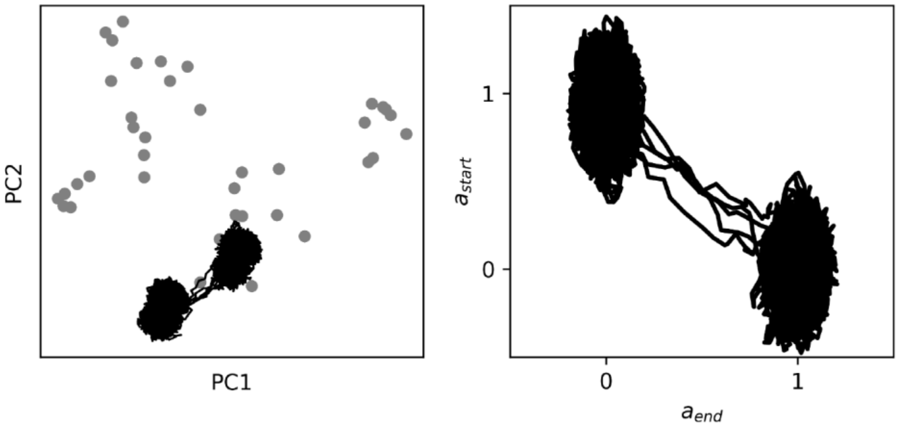


\subsection*{Figure S2. Stochastic reprogramming dynamics (Related to Figure 2).}

Here we considered the stochastic dynamics during reprogramming by using the stochastic version of Eq.~\ref{eq:enhancernetsym} (Methods), setting $\sigma=0.02$, and a modified reprogramming weight of $\delta=0.85$ for the TF \emph{Ascl1}. The initial condition is set to the fibroblast state. Here, the transition from fibroblast to neuron is not deterministic, but occurs stochastically as a memoryless barrier crossing. The left panel displays the dynamics after PCA transformation on all the cell types, while the right panel displays the dynamics transformed by an orthogonal projection of the TF profiles of the tabula muris cell types, as in \cite{lang2014epigenetic,pusuluri2017cellular}. Here $a_{\text{start}}$ corresponds to projection of the dynamics on the fibroblast cell fate and $a_{\text{end}}$ corresponds to the projection on the neuron state.

I found the discussion of annealing temperature interesting. It is a property of all Hopfield like models that the low energy ``spurious attractor" are always mixtures of existing cell types, and more correlated they are, the more likely the spurious attractors (see discussion in Amit's book Modeling Brain Function for nice summary of the literature). However, in earlier works such as Lang et al. it was argued that such attractors are likely not biologically meaningful but instead correspond to in vitro objects such as partially reprogrammed cells. I found the proposal here that these spurious attractors may correspond to progenitors quite interesting. The clustering in Figure 3c essentially builds a tree between closely related cells. However, recent lineage tracing experiments (Weinreb et al. Science 367.6479 (2020)) indicate that the picture might be more complicated, including progenitors that can give rise to three or more cell types. It was unclear if the model captures this more refined information. Also, in other lineages, I suspect that the model may predict progenitors between all closely related cell types? Are there scenarios where there is high cosine-similarity but no progenitor state? How do you reconcile this with biological experiments if there is not?

We indeed propose that the model can explain the existence, identity, and differentiation properties of progenitor states with multilineage priming, such as the well-characterised progenitor states in haematopoiesis. A key distinguishing feature of our model is that these attractor states can be stabilised and destabilised by the cell through modulation of ß (through e.g. modulation of global histone acetylation rates). We specifically propose that differentiation proceeds through an annealing process whereby the energy landscape is continuously modulated until there is convergence to an induced cell type. The existence of progenitor cell states can in a sense be considered a byproduct of this search process. We revised our discussion and the relevant results section to clarify this.

The model can also explain complex differentiation patterns, including progenitors that can differentiate into multiple cell types. The tree in Figure 1C is generated by hierarchical clustering and is useful for visualising the correlation structure in the data, however, differentiation trajectories do not need to be tree-like, as we show in Figure 1D (which shows simulations of differentiation trajectories in haematopoiesis). It is possible to have progenitors that differentiate into three cell types – this would occur if these have correlated expression patterns

© 2024. Published by The Company of Biologists under the terms of the Creative Commons Attribution License (https://creativecommons.org/licenses/by/4.0/).

that are distinct from other cell types. The progenitors observed by Weinreb et al. are entirely consistent with this. For example, we can see in Figure 1D that differentiation to neutrophils occurs in the model through distinct macrophage-neutrophil and neutrophil-eosinophil-basophil progenitors, as observed in the analysis of the data from Weinreb et al. by Yampolskaya et al. To improve the presentation of this, we have now included a new supplementary figure (Figure S4) which demonstrates proliferation through tripotent progenitors and deviations from tree-like hierarchy for simple gene expression patterns.

Finally, regarding the progenitor prediction, indeed the model predicts that in the case where there is differentiation from multipotent progenitors to multiple cell fates, then progenitors would preferentially appear near similar terminal fates (the precise criteria is slightly more complex as it depends on other patterns, as can be seen in the example from the previous paragraph). Adult haematopoiesis is a good system to test this as it is (a) complex, with a single stem/progenitor giving rise to multiple cell fates, (b) well-characterised in terms of the identity of the progenitor states and the existence of multilineage priming, and (c) occurs at steady state, so we can assume that the dynamics are fixed (unlike developmental settings). While complex patterns of differentiation in other tissues are, at the moment, not as well characterised, our prediction also appears to be supported by a recent study on differentiation in the intestine (now discussed in the revised manuscript) [Singh et al. 2022]. In this study, the authors identified differentiation through multilineage priming in the intestine epithelium, with multipotent progenitors for the secretory cells and bipotent progenitors for Paneth/Goblet cells and between endocrine cell types, which is precisely the structure anticipated from the similarities between the cell types.

The revised Results section presenting the hematopoiesis analysis:

The model makes the specific prediction that differentiation hierarchies, including the identity of observed progenitor states, can be estimated by only knowing the identities of the terminal cell states. The model specifically predicts that they will appear only between transcriptional profiles with high internal cosine similarity. To test this, we considered differentiation in hematopoiesis, a system in which differentiation trajectories are complex and have been extensively studied. Using data on hematopoietic lineages in mice (extracted from Haemopedia \cite{choi2019haemopedia}), we considered the expression profile of all TFs that showed variability between terminal lineages and used the model to estimate the differentiation hierarchy that generated these lineages.

We first tested the hypothesis that progenitors emerge between correlated expression profiles by performing hierarchical clustering according to pairwise cosine similarity between the expression profiles of terminal cell types (Figure 3C). Hierarchical clustering provides a heuristic approach to estimate the predicted identity of progenitor states from the terminal expression profiles. It produces a dendrogram (tree) that shows how expression profiles are progressively grouped into clusters based on their similarity. This resulted in a tree structure that recreates the classic hematopoietic hierarchy (Figure 3C). Branching points at the dendrogram capture the Common Lymphoid Progenitor (CLP), Common Myeloid Progenitor (CMP), Megakaryocyte and Erythroid Progenitor (MEP), Granulocyte and Macrophage Progenitor (GMP), T and NK cell progenitors (TNK), and B cell-biased lymphoid progenitor (BLP). Thus, a simple unsupervised clustering method with access to only the terminal cell fate information can reproduce a faithful estimate of the known complex differentiation landscape in hematopoiesis.

Motivated by the effectiveness of hierarchical clustering in predicting progenitor states, we developed a computational approach to model differentiation into multiple cell types. This approach is based on annealing in Eq.~\ref{eq:enhancernetsym}. We simulated annealing trajectories beginning with a homogeneous initial population representing hematopoietic stem cells. To account for natural variability in differentiation paths, we introduced noise to Equation \ref{eq:enhancernetsym} (our findings remain robust across various noise magnitudes, Figure S3). In physiological settings, the production rate of each cell type is regulated by negative feedback, where the terminal cell population size inhibits its own production. This is exemplified by the negative feedback regulation of red blood count through EPO signalling \cite{grover2014erythropoietin}. To incorporate this phenomenon, we implemented a negative feedback routine to tune $\textbf{w}$, allowing each cell type to inhibit its own production

© 2024. Published by The Company of Biologists under the terms of the Creative Commons Attribution License (https://creativecommons.org/licenses/by/4.0/).

(Methods). This resulted in dynamics that generated all cell fates in a balanced manner. We then visualized the differentiation trajectories on a UMAP plot (Figure 3D), revealing that differentiation dynamics proceed through a series of multipotent progenitors corresponding to those observed in hematopoiesis. Thus, our model quantitatively recapitulates differentiation in hematopoiesis without requiring parameter fitting.

While the heuristic approach of hierarchical clustering produces a tree-like structure that recapitulates the main progenitor states in hematopoiesis, differentiation trajectories in the model need not be tree-like, as can be seen in the simulations of Figure 3D. As $\beta$ increases, multiple steady states can appear and disappear, and these new steady states may be averages of overlapping terminal states. The key prediction of the model is that these states will correspond to averages of terminal states with high internal similarity. For example, it is experimentally established that dendritic cells can emerge from both the myeloid lineage through macrophage-dendritic progenitors and from the lymphoid lineage through B cell-biased lymphoid progenitors \cite{anderson2021genetic}. This observation aligns with our model and simulations (Figure 3D, see also Figure S4 for simple examples of tripotent progenitors and deviations from tree structure). The gene expression profile of dendritic cells exhibits high cosine similarity to both B cells and macrophages (and, more generally, myeloid cells), while B cells are less similar to myeloid cells. Similarly, neutrophils may arise from distinct macrophage-neutrophil and basophil-eosinophil-neutrophil progenitors, and macrophages from distinct macrophage-neutrophil and macrophage-dendritic cell progenitors \cite{weinreb2020lineage,yampolskaya2023sctop}. These patterns are also recapitulated in our simulations and are related to the similarity structure of the cell types. Higher-level progenitor structures can be captured in the same manner by the model. For example, lymphoid and granulocyte cells have moderate cosine similarity, as do granulocyte and erythro-megakaryocytes. However, lymphoid and erythro-megakaryocytes are poorly correlated. Consequently, each of the first two pairs can share accessible progenitors, while the latter is less likely to do so. Indeed, the first two progenitors exist (Lympho-Myeloid Primed Progenitors and Common Myeloid Progenitors), while the latter, to the best of our knowledge, does not. The model can thus make predictions regarding highly complex transition dynamics by only using information on the terminal cell identities.

The new Discussion paragraph discussing differentiation in the intestinal epithelium:

For differentiation, the model predicts the identity of progenitors and can predict complex differentiation hierarchies by using only the identities of the terminal cell types. Specifically, the model predicts that in cases where a single multipotent progenitor gives rise to multiple cell types, more restricted progenitors will preferentially appear between sets of cell types with similar expression profiles that are distinct from other cell types. Our model recapitulates the complex and well-characterized differentiation hierarchy of hematopoiesis with no fitting parameters. In another well-studied system, intestinal stem cells differentiate into secretory progenitors, which then give rise to specific progenitors for Paneth/Goblet cells and Enterochromaffin/Non-enterochromaffin enteroendocrine cells \cite{singh2022cell}. Singh et al. showed that the progenitors exhibit multilineage priming at both the transcriptional level, with low-level expression of cell-type specific transcripts, and at the chromatin level, with intermediate chromatin accessibility signatures at cell-type specific enhancers. The identity of these progenitors, along with their epigenetic and transcriptional profiles, aligns closely with the model predictions that are based on the similarities among these cell types.

The new Figure S4:

© 2024. Published by The Company of Biologists under the terms of the Creative Commons Attribution License (https://creativecommons.org/licenses/by/4.0/).

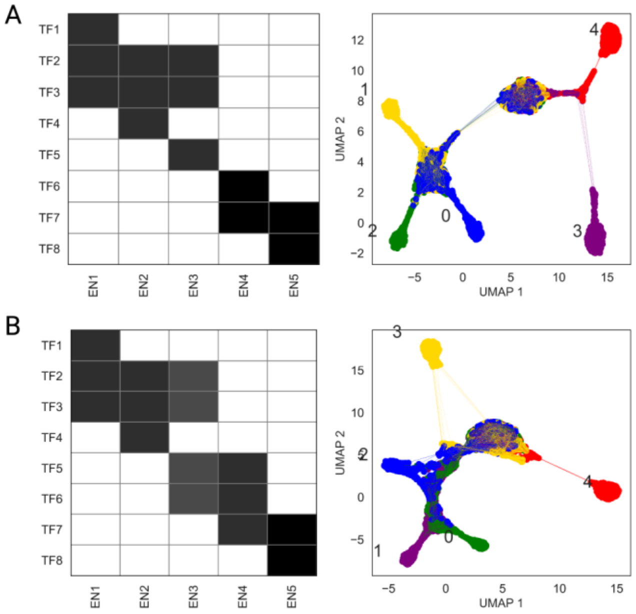


\subsection*{Figure S4. Tripotent and bipotent progenitors (Related to Figure 3).}

(A) Differentiation by annealing, where the first three cell types and the last two cell types have highly correlated expression patterns. Differentiation proceeds through a tripotent progenitor for the first set of cell types, and a bipotent progenitor for the latter. (B) Differentiation by annealing, where there is a complex correlation structure, can result in deviation from tree-like structure with multiple paths for differentiation to the terminal cell types. In all simulations, we used $\sigma=0.05$ and a maximum annealing inv. temperature of $\beta=12$.

For the evolution question, I think the Modern Hopfield networks are much more stable than traditional Hopfield networks because of the exponential storage capacity. However, it is unclear to me if this evolution also requires temperature (beta) to be extremely small. It would be nice to understand how the ability to evolve depends on the choice of temperature and the loading of these networks?

To address this issue, I have added a new methods section titled "Temperature transitions for cell type evolution." In this section, I derive the β required for stabilising evolved cell types as a function of the loading and the number of TFs in the network.

I specifically considered a "gain" in β, defined as the ratio between the β required to stabilise evolved cell types and the β required to destabilise a multipotent progenitor of related cell types. For example, this could be the β required to stabilise a new type of neuron or blood cell, compared to the β required to destabilise a common progenitor for all neurons or all blood cells.

I demonstrate that this gain is given by the ratio of the variance in the number of active TFs among related cell types to the logarithm of the number of related cell types. As a concrete case study, I estimated the gain for C. elegans neurons, where data is available to extract the relevant parameters. I identified an 80% gain in β required from the destabilisation of a neural multipotent progenitor to the full specification of all neuronal identities, including discrimination between closely related neural types.

In general, the dependence of the gain on the parameters is mild, and I do not expect that it would need to be extremely large for the specification of new cell types.

The revised Results section on cell type evolution:

We conclude this study by considering the question of how new cell types can evolve within the regulatory network? Although in principle a new cell type may arise \emph{de novo}, in practice it is likely to appear by the diversification of an ancestral cell type into sister cell types, a process

© 2024. Published by The Company of Biologists under the terms of the Creative Commons Attribution License (https://creativecommons.org/licenses/by/4.0/).

known as genetic individuation \cite{arendt2016origin,hobert2021homeobox}. In this process, a cell type associated with a specific combination of TFs evolves into new and distinct cell types associated with new TF combinations.

The EnhancerNet model has unique properties that support the process of genetic individuation of cell types; we propose that these properties may have been instrumental for the evolution of distal cis-regulatory elements with dynamic chromatin in animals. These properties are (i) the ability to support multiple coexisting, highly correlated attractor states, and (ii) the ability to have multiple enhancers regulating the same genes.

The importance of the first property is clear when we consider that around the time of the initial diversification of the ancestral cell types, the sister cells have highly correlated expression patterns. At this stage, they need to coexist as distinct attractor states to be expressed in the body. Thus, in the biological context, the ability to support multiple correlated attractor cell type states is crucial. This is supported by the mechanism underlying the EnhancerNet through the inverse temperature parameter $\beta$, which allows cells to discriminate between highly similar expression patterns.

As a concrete example, consider the specification of neuronal identity in C. elegans worms. Reilly et al. investigated the expression of TFs in the mature worm nervous system \cite{reilly2020unique}. Their findings showed that each of the 118 neuron classes in C. elegans is defined by a unique combination of homeobox TFs, with 68 TFs exhibiting variable expression across neuronal classes. Many neuronal classes share highly similar TF codes: 8 classes differ from another class by only a single TF, while 70 classes differ from another by 3 or fewer TFs. These highly correlated expression patterns pose a challenge for classic attractor models networks, which cannot co-stabilize them. However, the mechanism underlying Eq.~\ref{eq:enhancernetsym}, through regulation of the $\beta$ parameter, successfully supports these patterns, with around a 2-fold increase in $\beta$ allowing full specification of all neuronal classes from a single multipotent progenitor (Methods).

The new Methods section:

\subsection*{Temperature transitions for cell type evolution}

In this section, we will estimate the temperature $\beta$ required to specify a population of related cell types. These cell types may share activity patterns across many of their TFs and differ in some smaller portion. As an example that will be discussed later, neurons may have similar TF activity patterns across many TFs, yet small differences in TF activity can fine-tune the identity of specific neurons.

We will specifically consider $K$ patterns associated with closely related cell types and $K'$ patterns associated with other cell types. There are $N$ bits that differ between the related cell types, of which a fraction $\eta$ are active (drawn at random), $N''$ bits that are inactive, and $N'$ bits that are active in all other cell types. Since patterns have magnitude unity, we set the activity level of active bits to $a=\frac{1}{\sqrt{\eta N+N'}}$. We assume that unrelated patterns have $\eta N + N'$ active TFs drawn at random.

The average inner product of the related cell types is given by:
\begin{equation}
A=\frac{\eta^2 N + N'}{\eta N + N'}
\end{equation}
While the average inner product between random cell types is given by:
\begin{equation}
B = \frac{\eta N + N'}{N + N'' + N'}
\end{equation}
Let $\Xi_1,\dots,\Xi_{K}$ denote each of the closely related patterns, and $\bar{\Xi}$ their average.
The energy of each pattern is given by:
\begin{equation}
V_1 = V(\Xi_1) \approx -\beta^{-1}\log{\left(e^{\beta} + (K-1){e^{\beta A} + K' e^{\beta B} }\right)} + \frac{1}{2}

© 2024. Published by The Company of Biologists under the terms of the Creative Commons Attribution License (https://creativecommons.org/licenses/by/4.0/).

\end{equation}
We will assume that the patterns are sufficiently well separated ($A\gg B$) and $\beta$ sufficiently large such that:
\begin{equation}
V_1 \approx -\beta^{-1}\log{\left(e^{\beta} + (K-1){e^{\beta A} }\right)} + \frac{1}{2}
\end{equation}
while the energy of the averaged pattern is:
\begin{equation}
V_2=V(\bar{\Xi})\approx -\beta^{-1}\log{K} - \frac{A}{2}
\end{equation}

In general, as $\beta$ increases, we expect to see first a transition from the global average to the local average of the two patterns, which occurs when $V_1$ drops below $V_2$. This transition occurs at a critical $\beta$ that satisfies (assuming $K\gg 1)$:
\begin{equation}
-\frac{1}{\beta} \log \left(e^{\beta (A-1)}+\frac{1}{K}\right)=\frac{1}{2}-\frac{A}{2}
\end{equation}
When $\beta$ is small then $e^{\beta (A-1)}\gg\frac{1}{K}$ and the l.h.s is equal to $1-A$ and thus larger than the r.h.s. When $e^{\beta (A-1)}\ll\frac{1}{K}$ the l.h.s is negative and thus smaller than the r.h.s. Thus, the critical $\beta$ occurs around:
\begin{equation}
\beta_{\text{crit}_1} \approx \frac{1}{1-A}\log{K} = \frac{1}{1-\eta } \left(\frac{N'}{\eta N}+1 \right)\log{K}
\label{eq:critA}
\end{equation}

We will now consider the case where a new cell type $\Xi_1'$ evolves from $\Xi_1$. We will assume, without loss of generally, that the evolved pattern $\Xi_1'$ differs from the existing pattern $\Xi_1$ by two bits, namely $\xi_{1,1}=a,\xi_{1,1}'=0,\xi_{1,2}=0,\xi_{1,2}'=a$.
The energy of the new patterns is given by:

\begin{equation}
V_3=V(\Xi_1')= V(\Xi_1) \approx -\beta^{-1}\log{\left(e^{\beta}+e^{\beta C}+ (K-1){e^{\beta A} }\right)} + \frac{1}{2}
\end{equation}
with $C$ corresponding to the inner product of the new cell types:
\begin{equation}
C = 1-\frac{1}{\eta N + N'}
\end{equation}
Whereas the energy of the averaged pattern is:
\begin{equation}
V_4 = V\left(\frac{1}{2}\left(\Xi_1'+\Xi_1\right)\right) \approx -\beta^{-1}\log{\left(2 e^{\beta C}+(K-1){e^{\beta A} }\right)} + \frac{C}{2}
\end{equation}
For the second transition, we are interested in $\beta>\beta_{\text{crit}_1}$ where the contributions of the $(K-1){e^{\beta B}}$ terms to the energy levels are negligible. In this case, we can use Eq.~\ref{eq:bipotent}:
\begin{equation}
\beta_{\textbf{crit}_2} = (\eta N + N') \log{4}
\label{eq:bipotentB}
\end{equation}
Using Eqs.~\ref{eq:bipotentB},\ref{eq:critA} we can derive a ratio $\chi$ that captures the relative increase in $\beta$ required to evolve and stabilize a new cell type from an existing population of cells:
\begin{equation}
\chi= \frac{\beta_{\textbf{crit}_2}}{\beta_{\textbf{crit}_1}} = \frac{(1-\eta ) \eta N \log{4}}{\log{K}}
\end{equation}
the gain $\chi$ thus only depends on the TFs that are variable between the cells, and, amongst these TFs, scales with the variance of the active TFs.

© 2024. Published by The Company of Biologists under the terms of the Creative Commons Attribution License (https://creativecommons.org/licenses/by/4.0/). 

For the case of neuronal identity specification in \emph{C. elegans}, the expression code is sparse, with $\mu=0.1$ among variable TFs, with the other parameters being $K=118$ neuron classes and $N=68$ TFs exhibiting variable expression across neuronal classes. For this system, we thus have that:
\begin{equation}
\chi= \frac{\beta_{\textbf{crit}_2}}{\beta_{\textbf{crit}_1}} = \frac{(1-\eta ) \eta N \log{4}}{\log{K}} = 1.8
\end{equation}
implying a further 80\% increase in $\beta$ required from the initial destabilization of a multipotent progenitor to the complete specification of all cell types.

Finally, my intuition is that the authors constructions are actually robust to adding a significant amount of asymmetry to the model. One easy way to test this is to numerically just add noise so that $Q = Xi + Noise$ for different levels of noise and see if the attractors are still robust. This would show that one just needs approximate symmetry not exact symmetry, making the model more biophysically plausible.

Thank you for this suggestion. In the revised manuscript, I tested the robustness of our model to asymmetry by introducing up to 30% noise in the \Xi matrix, specifically considering the stability and identity of the fixed points after noising on the data from Figure 2 and on data drawn from a uniform distribution. The dynamics are indeed highly robust, and we now add the analysis in a new supplementary figure:

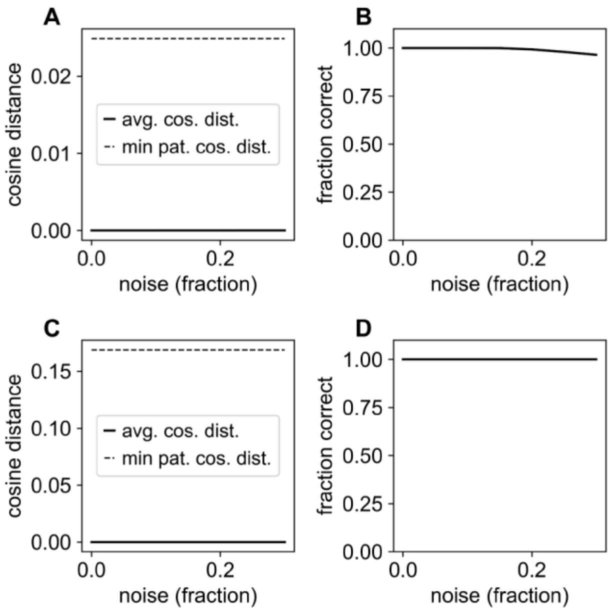


\subsection*{Figure S1. Robustness of fixed points to asymmetry in enhancer-TF interactions (Related to Figure 2).}

Throughout the manuscript, we assume symmetry in enhancer-TF interactions, captured mathematically by $\textbf{Q}=\Xi$. However, in practice, it is likely that the matrices are correlated but not perfectly identical, that is, there is some asymmetry in the dynamics. To test for the effect of this asymmetry, we added noise to the entries of $\Xi$ by multiplying each entry by a scalar drawn from a normal distribution with mean $1$ and standard deviation ranging from $0$ to $0.3$ (entries were capped at a minimum of $0$) while fixing the \textbf{Q} matrix. We used the $\Xi$ matrix based on the Tabula Muris data (A,B) or a matrix initialized with uniformly drawn entries of the same dimensions (C,D). As in Figure 2, the dynamics were initialized from each of the rows of $\textbf{Q}$. Even at very high noise magnitudes, the dynamics always settle at a cell type specified by the $\textbf{Q}$ matrix (A,C, solid line denoting average minimal cosine distance of final state from rows of $\textbf{Q}$, dashed line minimal cosine distance between rows of $\textbf{Q}$). The only effect of noising is to destroy specific attractors of very similar cell types, such as T/NK cells, by destroying their distinguishing features (B,D, line

© 2024. Published by The Company of Biologists under the terms of the Creative Commons Attribution License (https://creativecommons.org/licenses/by/4.0/).

capturing average fraction of final states that are identical to the initial state).

---

Second decision letter

MS ID#: DEVELOP/2024/202997

MS TITLE: EnhancerNet: A predictive model of cell identity dynamics through enhancer selection

AUTHORS: Omer Karin

I have now received all the referees reports on the above manuscript, and have reached a decision. The referees' comments are appended below, or you can access them online: please go to BenchPress and click on the 'Manuscripts with Decisions' queue in the Author Area.

The overall evaluation is very positive and we would like to publish a revised manuscript in Development, provided that the last comment of Referee 2 is satisfactorily addressed (which should not be difficult). If you do not agree with their criticisms or suggestions explain clearly why this is so.

Reviewer 1

*Advance summary and potential significance to field*

The author has now comprehensively revised the manuscript and most importantly improved the approachability of the presentation. The approximations inherent in the approach have also been laid out in a much more systematic manner. I am now happy for the manuscript to be published.

*Comments for the author*

No further suggestions

Reviewer 2

*Advance summary and potential significance to field*

Please see last review.

*Comments for the author*

I am very happy with the changes. I think the paper is ready to go. As I said last time, I think this is a very nice work.

I would say the one thing that needs to be changed is that the author states in multiple places in reference to classical Hopfield models of cell identity that they cannot store correlated patterns. For example, "Crucially, these networks cannot stabilize correlated cell types, which are inherently prevalent in bio- logical systems." This is INCORRECT.

Many of the cited papers (Lang, Pulsuri, Smart) actually use the projection method variant of the Hopfield model and hence CAN and DO store extremely correlated patterns. This is why they were used for cell identity. The capacity of this models is also N (where N is the number of TFs) so there is no really problem with capacity since there do not seem to be more cell types than TFs.

© 2024. Published by The Company of Biologists under the terms of the Creative Commons Attribution License (https://creativecommons.org/licenses/by/4.0/). 

However, as the author rightly points out, the main draw back of these classical models is that they require binarization whereas modern Hopfield models do not. This should be emphasized more and the correlation statements fixed.

---

**Second revision**

Author response to reviewers' comments

Dear Dr. Francois,

Thank you for sharing the referees' comments and for the favourable evaluation of the manuscript. I agree with the comment of referee 2 – while the previous manuscript version mentioned the limitations of the (unprojected) classical Hopfield dynamics for storing correlated patterns, these limitations can indeed be overcome by the projection method. I have now revised the manuscript throughout to account for this. I would like again to express my appreciation for the thorough and constructive review process.

Best regards,
Omer Karin

---

Third decision letter

MS ID#: DEVELOP/2024/202997

MS TITLE: EnhancerNet: A predictive model of cell identity dynamics through enhancer selection

AUTHORS: Omer Karin

I am happy to tell you that your manuscript has been accepted for publication in Development, pending our standard publication integrity checks.

© 2024. Published by The Company of Biologists under the terms of the Creative Commons Attribution License (https://creativecommons.org/licenses/by/4.0/).
